# Supplementary material for: Association of severe mental illness and septic shock case fatality rate in patients admitted to the intensive care unit: A national population-based cohort study
Source: PLoS Med. 2023 Mar 13;20(3):e1004202. doi: 10.1371/journal.pmed.1004202 (PMC10042353; doi:10.1371/journal.pmed.1004202)
Supplement: S1 STROBE Checklist — (DOCX) [file pmed.1004202.s001.docx]

STROBE Statement—Checklist of items that should be included in reports of ***cohort studies***

|  | Item No | Recommendation | Page No |
| --- | --- | --- | --- |
| **Title and abstract** | 1 | (*a*) Indicate the study’s design with a commonly used term in the title or the abstract | p1 |
|  |  | (*b*) Provide in the abstract an informative and balanced summary of what was done and what was found | p3 |
| Introduction | | | |
| Background/rationale | 2 | Explain the scientific background and rationale for the investigation being reported | p6-7,  Introduction section, §1 and 2 |
| Objectives | 3 | State specific objectives, including any prespecified hypotheses | p7, Introduction section, §3 |
| Methods | | | |
| Study design | 4 | Present key elements of study design early in the paper | p7,  Method section, § *Study design, sources, and population* |
| Setting | 5 | Describe the setting, locations, and relevant dates, including periods of recruitment, exposure, follow-up, and data collection | p7-8, Method section, § *Study design, sources, and population* and § *Collected data* |
| Participants | 6 | (*a*) Give the eligibility criteria, and the sources and methods of selection of participants. Describe methods of follow-up | p7 Method section, § *Study design, sources, and population* |
|  |  | (*b*) For matched studies, give matching criteria and number of exposed and unexposed | p9 Method section, § *Exposure* |
| Variables | 7 | Clearly define all outcomes, exposures, predictors, potential confounders, and effect modifiers. Give diagnostic criteria, if applicable | p8 Method section, § *Outcomes*, p9 Method section, § *Exposure* |
| Data sources/ measurement | 8* | For each variable of interest, give sources of data and details of methods of assessment (measurement). Describe comparability of assessment methods if there is more than one group | p7-8 Method section, § *Study design, sources, and population* and § *Collected data* |
| Bias | 9 | Describe any efforts to address potential sources of bias | p9 Method section, § *Statistical analysis* |
| Study size | 10 | Explain how the study size was arrived at | p7 Method section, § *Study design, sources, and population* |
| Quantitative variables | 11 | Explain how quantitative variables were handled in the analyses. If applicable, describe which groupings were chosen and why | p8-9 Method section, § *Statistical analysis* and § *Collected data* |
| Statistical methods | 12 | (*a*) Describe all statistical methods, including those used to control for confounding | p9 Method section, § *Statistical analysis* |
|  |  | (*b*) Describe any methods used to examine subgroups and interactions | p9 Method section, § *Statistical analysis* |
|  |  | (*c*) Explain how missing data were addressed | p9 Method section, § *Statistical analysis* |
|  |  | (*d*) If applicable, explain how loss to follow-up was addressed | p9 Method section, § *Statistical analysis* |
|  |  | (*e*) Describe any sensitivity analyses | p9 Method section, § *Statistical analysis* |
| Results | | |  |
| Participants | 13* | (a) Report numbers of individuals at each stage of study—eg numbers potentially eligible, examined for eligibility, confirmed eligible, included in the study, completing follow-up, and analysed | p10, Result section, first § |
|  |  | (b) Give reasons for non-participation at each stage | NA |
|  |  | (c) Consider use of a flow diagram | p10-Fig.1 |
| Descriptive data | 14* | (a) Give characteristics of study participants (eg demographic, clinical, social) and information on exposures and potential confounders | p10-11 Result section, first § |
|  |  | (b) Indicate number of participants with missing data for each variable of interest | NA |
|  |  | (c) Summarise follow-up time (eg, average and total amount) | NA |
| Outcome data | 15* | Report numbers of outcome events or summary measures over time | p10, Result section, first § |

| Main results | 16 | (*a*) Give unadjusted estimates and, if applicable, confounder-adjusted estimates and their precision (eg, 95% confidence interval). Make clear which confounders were adjusted for and why they were included | p10-11, Result section, all § |
| --- | --- | --- | --- |
|  |  | (*b*) Report category boundaries when continuous variables were categorized | p10, Result section, first § |
|  |  | (*c*) If relevant, consider translating estimates of relative risk into absolute risk for a meaningful time period | NA |
| Other analyses | 17 | Report other analyses done—eg analyses of subgroups and interactions, and sensitivity analyses | p11, Result section, second and third § |
| Discussion | | | |
| Key results | 18 | Summarise key results with reference to study objectives | p11, Discussion section, first § |
| Limitations | 19 | Discuss limitations of the study, taking into account sources of potential bias or imprecision. Discuss both direction and magnitude of any potential bias | p13, Discussion section, fifth § |
| Interpretation | 20 | Give a cautious overall interpretation of results considering objectives, limitations, multiplicity of analyses, results from similar studies, and other relevant evidence | p11-13, Discussion section, all § |
| Generalisability | 21 | Discuss the generalisability (external validity) of the study results | p13-14, Discussion section, second, third fourth and fifth § |
| Other information | | | |
| Funding | 22 | Give the source of funding and the role of the funders for the present study and, if applicable, for the original study on which the present article is based | p15 |

*Give information separately for exposed and unexposed groups.

**Note:** An Explanation and Elaboration article discusses each checklist item and gives methodological background and published examples of transparent reporting. The STROBE checklist is best used in conjunction with this article (freely available on the Web sites of PLoS Medicine at http://www.plosmedicine.org/, Annals of Internal Medicine at http://www.annals.org/, and Epidemiology at http://www.epidem.com/). Information on the STROBE Initiative is available at http://www.strobe-statement.org.
