# Supplementary material for: Association of severe mental illness and septic shock case fatality rate in patients admitted to the intensive care unit: A national population-based cohort study
Source: PLoS Med. 2023 Mar 13;20(3):e1004202. doi: 10.1371/journal.pmed.1004202 (PMC10042353; doi:10.1371/journal.pmed.1004202)
Supplement: S1 Fig — (DOCX) [file pmed.1004202.s002.docx]

**S1 Fig.** Forest plots of unadjusted (model 1) and adjusted hazard ratios (main model and sensitivity analyses) for 90-day hospital septic shock case fatality in septic shock patients with severe mental illness compared to those without*


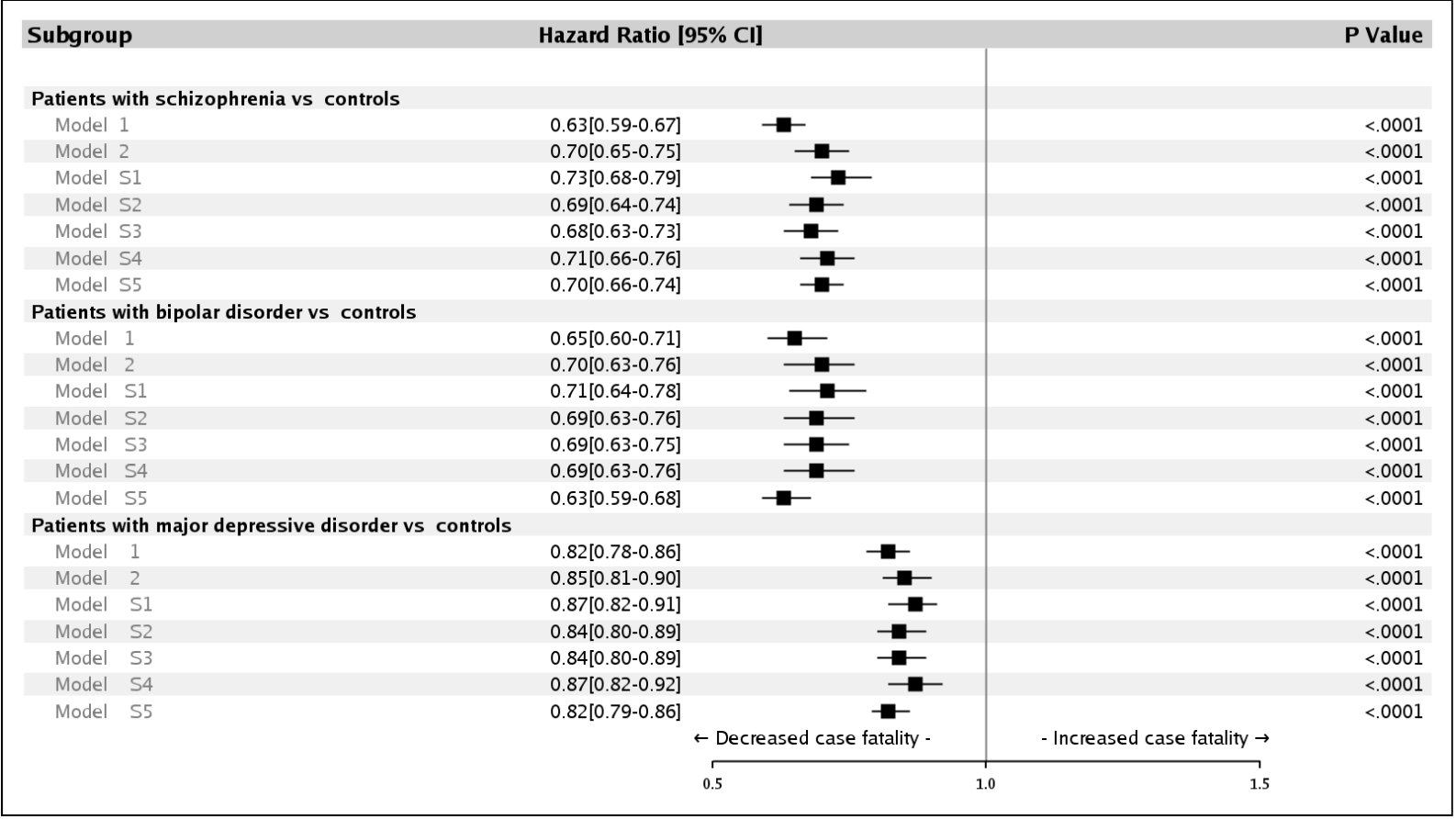


** 1: up to 4 patients matched, within a hospital, for age (5-year range), sex, degree of social deprivation, and year of hospitalization.*

*Square: hazard ratio, Line: 95% confidence interval (95% CI).*

***Model 1*** *included SMI only (without adjustment);* ***Model 2*** *included SMI with additional covariates of smoking, alcohol, and other substance addiction (yes vs. no), overweight or obesity (yes vs. no), the Charlson comorbidity index (0, 1-2, ≥3), presence of trauma (yes vs. no), surgical intervention (yes vs. no), SAPS II score (modified, without age), organ failures (yes vs. no for each of respiratory, renal, neurologic, cardiovascular, hematologic, metabolic, hepatic), the source of hospital admission (home, transfer from other hospital ward) and time to ICU admission (≤1 vs. > 1 day);* ***Model S1*** *is the model 2 with the 17 Charlson comorbidities instead of the Charlson comorbidity index;* ***Model S2*** *is model 2 with the infected organs instead of the organ failures;* ***Model S3*** *is model 2 with the ICU supportive therapies instead of the organ failures;* ***Model S4*** *is model 2 with the nature of isolated pathogens;* ***Model S5*** *is performed on the whole cohort (without matching process) using the same variables as in model 2 and matching variables to consider residual bias from incomplete matching of controls to the respective SMI group.*
