# Supplementary material for: Association of severe mental illness and septic shock case fatality rate in patients admitted to the intensive care unit: A national population-based cohort study
Source: PLoS Med. 2023 Mar 13;20(3):e1004202. doi: 10.1371/journal.pmed.1004202 (PMC10042353; doi:10.1371/journal.pmed.1004202)
Supplement: S4 Fig — (A) Overall survival in septic shock patients with schizophrenia compared to matched controls without severe mental illness. (B) Overall survival in septic shock patients with bipolar disorder compared to matched controls without severe mental illness. (C) Overall survival in septic shock patients with major depressive disorder compared to matched controls without severe mental illness. (DOCX) [file pmed.1004202.s005.docx]

**S4 Fig.** Kaplan–Meier estimates of overall survival at one year after intensive care unit (ICU) admission in septic shock patients with and without severe mental illness*

A. Overall survival in septic shock patients with schizophrenia compared to matched controls without severe mental illness*


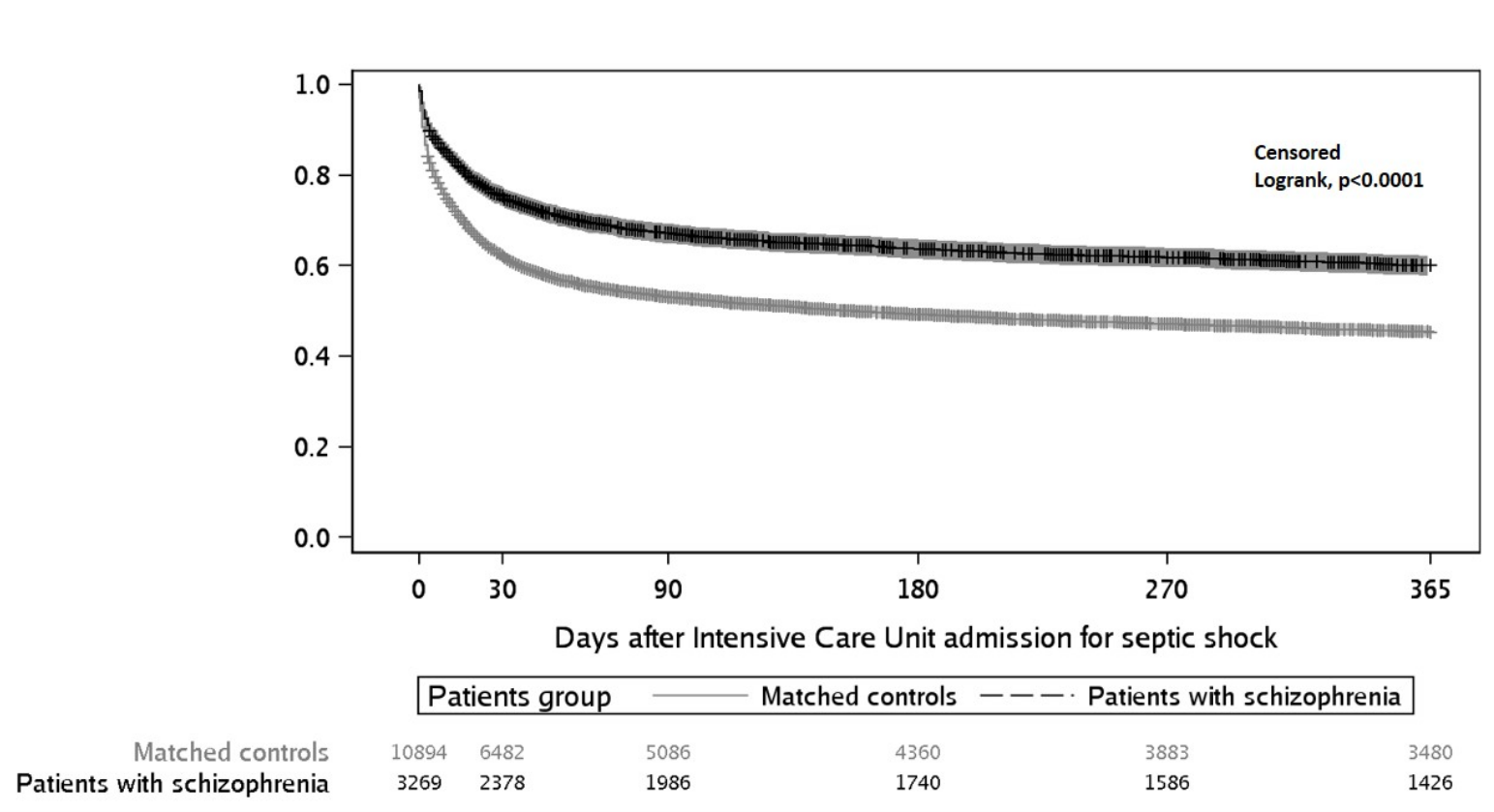


** 1:up to 4 patients matched, within a hospital, for age (5-year range), sex, degree of social deprivation, and year of hospitalization.*

B. Overall survival in septic shock patients with bipolar disorder compared to matched controls without severe mental illness*.


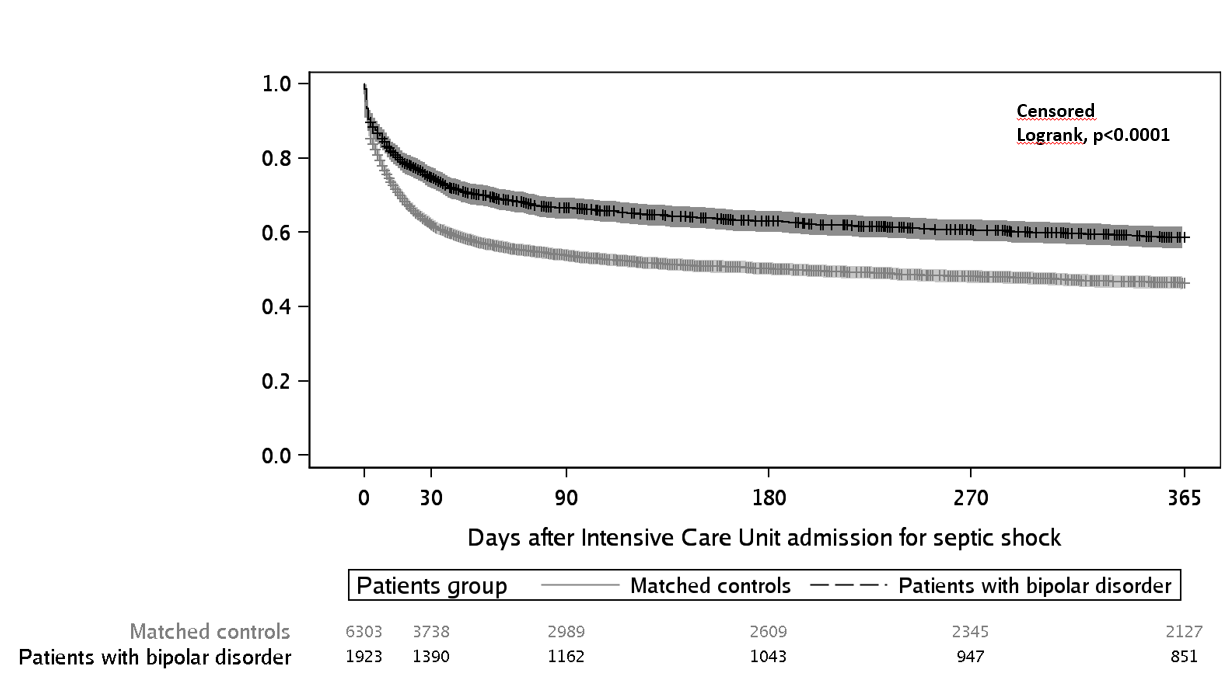


** 1: up to 4 patients matched, within a hospital, for age (5-year range), sex, degree of social deprivation, and year of hospitalization.*

C. Overall survival in septic shock patients with major depressive disorder compared to matched controls without severe mental illness*


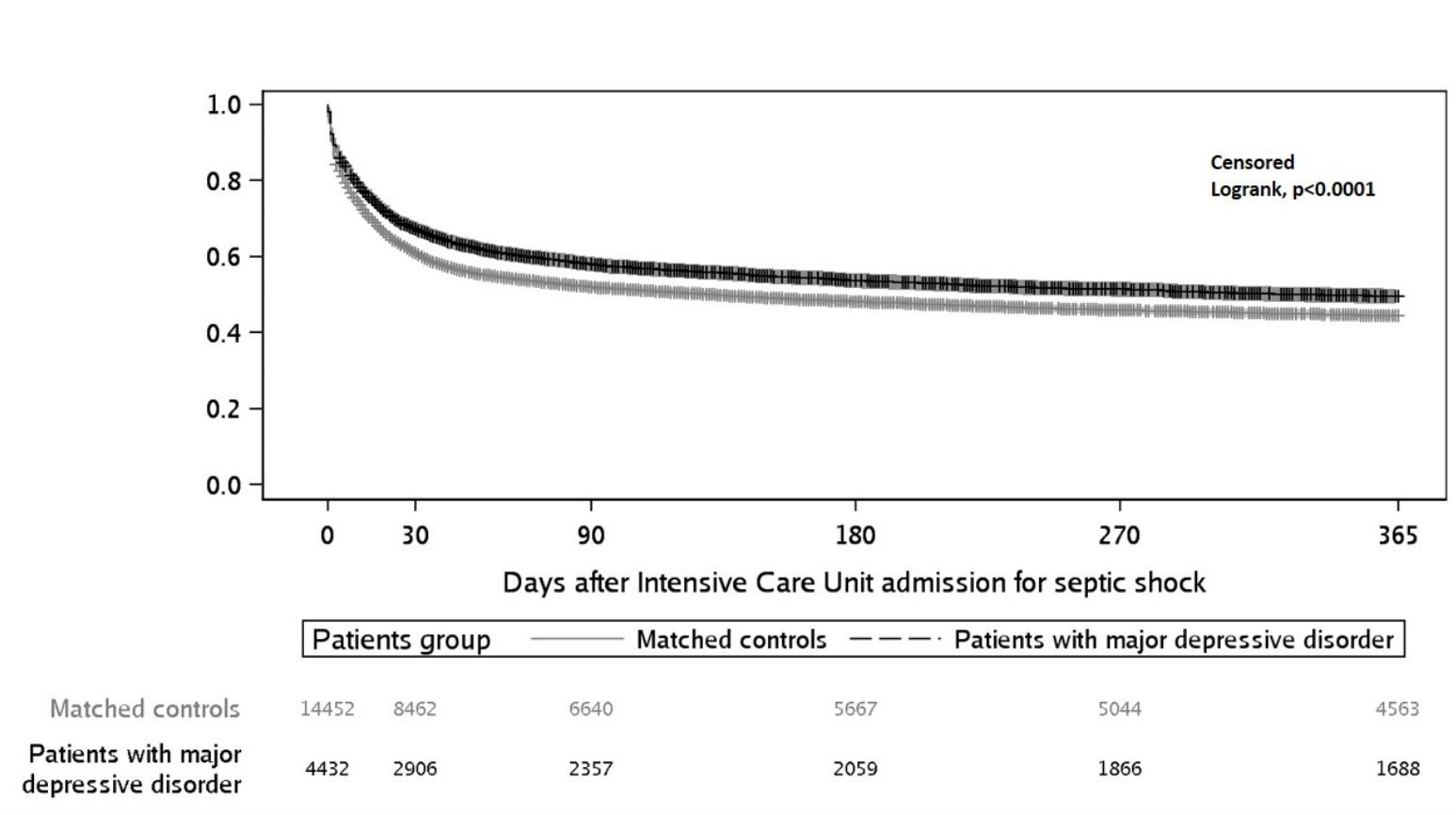


** 1: up to 4 patients matched, within a hospital, for age (5-year range), sex, degree of social deprivation and year of hospitalization.*
