# Supplementary material for: Association of severe mental illness and septic shock case fatality rate in patients admitted to the intensive care unit: A national population-based cohort study
Source: PLoS Med. 2023 Mar 13;20(3):e1004202. doi: 10.1371/journal.pmed.1004202 (PMC10042353; doi:10.1371/journal.pmed.1004202)
Supplement: S1 Table — (DOCX) [file pmed.1004202.s006.docx]

**S1 Table.** Charlson comorbidities of septic shock patients with and without severe mental illness*

|  | Patients with schizophrenia | Matched controls | SD† | p-value† | Patients with bipolar disorder | Matched controls | SD‡ | p-value‡ | Patients with major depressive disorder | Matched controls | SD⨎ | p-value⨎ |
| --- | --- | --- | --- | --- | --- | --- | --- | --- | --- | --- | --- | --- |
| N | 3,269 | 10,894 |  |  | 1,923 | 6,303 |  |  | 4,432 | 14,452 |  |  |
| Renal disease, n (%)  [95% CI] | 476  (14.6%)  [13.4-15.8] | 1,992  (17.6%)  [16.3-18.9] | -0.08 | **0.008** | 331  (17.2%)  [15.5-18.9] | 1,234  (18.8%)  [17.1-20.6] | -0.04 | 0.192 | 936  (21.1%)  [19.9-22.3] | 2,880 (19.3%)  [18.2-20.5] | 0.04 | **0.037** |
| Liver mild disease, n (%)  [95% CI] | 338  (10.3%)  [9.3-11.4] | 1,769  (16.3%)  [15.0-17.5] | -0.18 | **0.002** | 194  (10.1%)  [8.7-11.4] | 890  (14.4%)  [12.8-15.9] | -0.13 | **<0.001** | 811  (18.3%)  [17.2-19.4] | 1,985 (13.7%)  [12.7-14.7] | 0.13 | **<0.001** |
| Liver moderate / severe disease, n (%)  [95% CI] | 145  (4.4%)  [3.7-5.1] | 1,069  (9.8%)  [8.7-10.8] | **-0.21** | **<0.001** | 97  (5.0%)  [4.1-6.0] | 531  (8.5%)  [7.3-9.8] | -0.14 | **<0.001** | 436  (9.8%)  [9.0-10.7] | 1,198  (8.2%)  [7.3-9.0] | 0.06 | **0.006** |
| Peptic ulcer, n (%)  [95% CI] | 188  (5.8%)  [5.0-6.5] | 634  (5.9%)  (5.1-6.7] | -0.01 | 0.792 | 118  (6.1%)  [5.1-7.2] | 359  (5.6%)  [4.6-6.6] | 0.02 | 0.489 | 317  (7.2%)  [6.4-7.9] | 831  (5.6%)  [4.9-6.2] | 0.06 | **0.002** |
| Chronic obstructive pulmonary disease, n (%)  [95% CI] | 711  (21.8%)  [20.3-23.2] | 2,126  (19.2%)  [17.8-20.5] | 0.06 | **0.010** | 451  (23.5%)  [21.6-25.3] | 1,271  (20.0%)  [18.2-21.8] | 0.08 | **<0.001** | 1,264 (28.5%)  [27.2-29.8] | 3,082 (21.1%)  [19.9-22.3] | 0.17 | **<0.001** |
| Congestive heart failure, n (%)  [95% CI] | 821  (25.1%)  [23.6-26.6] | 3,446  (30.3%)  [28.8-31.9] | -0.12 | **<0.001** | 508  (26.4%)  [24.4-28.4] | 2,097  (32.4%)  [30.3-34.5] | -0.13 | **<0.001** | 1,534 (34.6%)  [33.2-36.0] | 5,105 (34.5%)  [33.1-35.9] | 0.00 | 0.927 |
| Myocardial infarction, n (%)  [95% CI] | 415  (12.7%)  [11.6-13.8] | 1,782  (15.4%)  [14.1-16.6] | -0.08 | **0.002** | 226  (11.8%)  [10.3-13.2 ] | 1,023  (15.4%)  [13.8-17.0] | -0.11 | **<0.001** | 756  (17.1%)  [16.0-18.2] | 2,479 (16.4%)  [15.3-17.5] | 0.02 | 0.430 |
| Peripheral vascular disease, n (%)  [95% CI] | 354  (10.8%)  [20.3-23.2] | 1,576  (13.8%)  [12.6-20.5] | -0.10 | **<0.001** | 186  (9.7%)  [8.4-11.0] | 910  (13.8%)  [12.3-15.4] | -0.13 | **<0.001** | 686  (15.5%)  [14.4-16.5] | 2,157 (14.2%)  [13.1-15.2] | 0.04 | 0.079 |
| Cerebrovascular disease, n (%)  [95% CI] | 335  (10.3%)  [9.2-11.3] | 1,448  (13.0%)  [11.9-14.2] | -0.09 | **<0.001** | 210  (10.9%)  [9.5-12.3] | 797  (12.5%)  [11.0-14.0] | -0.05 | 0.130 | 643  (14.5%)  [13.5-15.5] | 1,907 (12.9%)  [11.9-13.9] | 0.05 | **0.031** |
| Dementia, n (%)  [95% CI] | 241  (7.4%)  [6.5-8.3] | 217  (2.0%)  [1.5-2.4] | **0.26** | **<0.001** | 144  (7.5%)  [6.3-8.7] | 169  (2.8%)  [2.1-3.6] | **0.21** | **<0.001** | 325  (7.3%)  [6.6-8.1] | 459  (3.2%)  [2.7-3.7] | 0.19 | **<0.001** |
| Hemi/paraplegia, n (%)  [95% CI] | 298  (9.1%)  [8.1-10.1] | 1,214  (11.0%)  [9.9-12.1] | -0.07 | **0.012** | 171  (8.9%)  [7.6-10.2] | 678  (10.8%)  [9.5-12.2] | -0.07 | **0.043** | 515  (11.6%)  [10.7-12.6] | 1,460 (10.2%)  [9.3-11.1] | 0.05 | **0.031** |
| Rheumatic disease, n (%)  [95% CI] | 33  (1.0%)  [0.7-1.4] | 257  (2.4%)  [1.8-2.9] | -0.11 | **<0.001** | 29  (1.5%)  [1.0-2.1] | 160  (2.5%)  [1.8-3.2] | -0.07 | **0.025** | 167  (3.8%)  [3.2-4.3] | 415  (3.0%)  [2.5-3.5] | 0.04 | **0.044** |
| Metastatic solid tumor, n (%)  [95% CI] | 166  (5.1%)  [4.3-5.8] | 1,161  (10.8%)  [9.7-11.8] | **-0.21** | **<0.001** | 98  (5.1%)  [4.1-6.1] | 713  (10.9%)  (9.5-12.3] | **-0.21** | **<0.001** | 331  (7.5%)  [6.7-8.2] | 1,505 (10.4%)  [9.5-11.3] | -0.10 | **<0.001** |
| Malignancy, n (%)  [95% CI] | 472  (14.4%)  [13.2-15.6] | 3,056  (27.4%)  [25.9-28.9] | **-0.32** | **<0.001** | 350  (18.2%)  [16.5-19.9] | 1,923  (29.3%)  [27.3-31.4] | **-0.26** | **<0.001** | 1,008 (22.7%)  [21.5-24.0] | 4,059 (27.3%)  [26.0-28.6] | -0.11 | **<0.001** |
| Complicated diabetes, n (%)  [95% CI] | 277  (8.5%)  [7.5-9.4] | 1,251  (11.0%)  [9.9-12.0] | -0.08 | **<0.001** | 180  (9.4%)  [8.1-10.7] | 684  (10.6%)  [9.2-11.9] | -0.04 | 0.209 | 615  (13.9%)  [12.9-14.9] | 1,685 (11.2%)  [10.2-12.1] | 0.08 | **<0.001** |
| Uncomplicated diabetes, n (%)  [95% CI] | 697  (21.3%)  [19.9-22.7] | 2,569  (22.6%)  [21.1-24.0] | -0.03 | 0.221 | 469  (24.4%)  [22.5-26.3] | 1,520  (23.9%)  [22.0-25.8] | 0.01 | 0.744 | 1,171 (26.4%)  [25.1-27.7] | 3,477 (24.0%)  [22.7-25.2] | 0.06 | **0.008** |
| AIDS/HIV, n (%)  [95% CI] | 62  (1.9%)  [1.4-2.4] | 205  (1.8%)  [1.4-2.3] | 0.01 | 0.801 | 23  (1.2%)  [0.7-1.7] | 83  (1.3%)  [0.8-1.7) | -0.00 | 0.883 | 79  (1.8%)  [1.4-2.2] | 160  (1.1%)  (0.8-1.4] | 0.06 | **0.004** |

** 1: up to 4 patients matched, within a hospital, for age (5-year range), sex, degree of social deprivation, and year of hospitalization.*

† *Standardized difference and p-value between patients with schizophrenia and matched controls;* ‡ *Standardized difference and p-value between patients with bipolar disorder and matched controls;* ⨎ *Standardized difference and p-value between patients with major depressive disorder and matched controls.*

*SD­≤|0.20| was chosen to indicate a negligible difference in the mean or prevalence of a variable between groups. SD>|0.20| shown in bold. P-value<0.05 shown in bold.*

*95% CI: 95% confidence interval.*
