# Supplementary material for: Association of severe mental illness and septic shock case fatality rate in patients admitted to the intensive care unit: A national population-based cohort study
Source: PLoS Med. 2023 Mar 13;20(3):e1004202. doi: 10.1371/journal.pmed.1004202 (PMC10042353; doi:10.1371/journal.pmed.1004202)
Supplement: S2 Table — (DOCX) [file pmed.1004202.s007.docx]

**S2 Table.** Pathogens in septic shock patients with and without severe mental illness*

|  | Patients with schizophrenia | Matched controls | SD† | p-value† | Patients with bipolar disorder | Matched controls | SD‡ | p-value‡ | Patients with major depressive disorder | Matched controls | SD⨎ | p-value⨎ |
| --- | --- | --- | --- | --- | --- | --- | --- | --- | --- | --- | --- | --- |
| n | 3,269 | 10,894 |  |  | 1,923 | 6,303 |  |  | 4,432 | 14,452 |  |  |
| Positive culture with identification, n (%)  [95% CI] | 2,254  (69.0%)  [67.4-70.5] | 7,293  (67.1%)  [65.5-68.7] | 0.04 | 0.105 | 1,274  (66.3%)  [64.1-68.4] | 4,221  (66.7%)  [66.7-68.8] | -0.01 | 0.756 | 3,059 (69.0%)  [67.7-70.4] | 9,540 (66.2%)  [64.8-67.6] | 0.06 | **0.005** |
| Multidrug resistant bacteria, n (%)  [95% CI] | 535  (16.4%)  [15.1-17.6] | 1,577  (14.3%)  [13.1-15.5] | 0.06 | **0.019** | 260  (13.5%)  [12.0-15.0] | 905  (14.1%)  [12.5-15.6] | -0.02 | 0.621 | 769  (17.4%)  [16.2-18.5] | 2,103 (14.8%)  [13.7-15.8] | 0.07 | **0.001** |
| Enterobacter, n (%)  [95% CI] | 1,187  (36.3%)  [34.7-38.0] | 3,500  (31.8%)  [30.2-33.4] | 0.10 | **<0.001** | 658  (34.2%)  [32.1-36.3] | 2,149  (34.1%)  [32.0-36.2] | 0.00 | 0.932 | 1,621 (36.6%)  [35.2-38.0] | 4,873 (34.2%)  [32.8-35.6] | 0.05 | **0.020** |
| Staphylococcus, n (%)  [95% CI] | 843  (25.8%)  [24.3-27.3] | 2,651  (24. 5%)  [23.0-25.9] | 0.03 | 0.212 | 478  (24.9%)  [22.9-26.8] | 1,480  (23.3%)  [21.4-25.2] | 0.04 | 0.257 | 1,189 (26.8%)  [25.5-28.1] | 3,318 (23.0%)  [21.8-24.3] | 0.09 | **<0.001** |
| Streptococcus, n (%)  [95% CI] | 699  (21.4%)  [20.0-22.8] | 2,300  (21.5%)  [20.1-22.9] | -0.00 | 0.928 | 380  (19.8%)  [18.0-21.5] | 1,302  (20.4%)  [18.6-22.2] | -0.02 | 0.636 | 887  (20.0%)  [18.8-21.2] | 3,013 (20.6%)  [19.4-21.8] | -0.02 | 0.472 |
| Pseudomonas aeruginosa, n (%)  [95% CI] | 498  (15.2%)  [14.0-16.5] | 1,605  (14.5%)  [13.2-15.7] | 0.02 | 0.370 | 275  (14.3%)  [12.7-15.9] | 840  (13.1%)  [11.6-14.6] | 0.04 | 0.267 | 676  (15.3%)  [14.2-16.3] | 2,005 (13.5%)  [12.5-14.5] | 0.05 | **0.020** |
| Candida, n (%)  [95% CI] | 415  (12.7%)  [11.6-13.8] | 1,212  (11.3%)  [10.2-12.4] | 0.04 | 0.090 | 232  (12.1%)  [10.6-13.5] | 758  (12.2%)  [10.8-13.7] | -0.00 | 0.882 | 577  (13.0%)  [12.0-14.0] | 1,679 (11.9%)  [10.9-12.8] | 0.03 | 0.102 |
| Hospital-acquired infection, n (%)  [95% CI] | 908  (27.8%)  [26.2-29.3] | 2,990  (27.4%)  [25.9-28.9] | 0.01 | 0.731 | 473  (24.6%)  [22.7-26.5] | 1,741  (27.0%)  [25.0-28.9] | -0.05 | 0.096 | 1,321 (29.8%)  [28.5-31.2] | 3,908 (26.9%)  [25.6-28.2] | 0.07 | **0.002** |

** 1: up to 4 patients matched, within a hospital, for age (5-year range), sex, degree of social deprivation, and year of hospitalization.*

† *Standardized difference and p-value between patients with schizophrenia and matched controls;* ‡ *Standardized difference and p-value between patients with bipolar disorder and matched controls;* ⨎ *Standardized difference and p-value between patients with major depressive disorder and matched controls.*

*SD­≤|0.20| was chosen to indicate a negligible difference in the mean or prevalence of a variable between groups. P-value<0.05 shown in bold.*

*95% CI: 95% confidence interval.*
