# Supplementary material for: Association of severe mental illness and septic shock case fatality rate in patients admitted to the intensive care unit: A national population-based cohort study
Source: PLoS Med. 2023 Mar 13;20(3):e1004202. doi: 10.1371/journal.pmed.1004202 (PMC10042353; doi:10.1371/journal.pmed.1004202)
Supplement: Attachment — Submitted filename: mental disorders in septic shock - PLOS Medicine.docx [file pmed.1004202.s008.docx]

The manuscript describes the association of three categories of mental disorders with mortality among ICU-managed patients with septic shock in a population-based cohort.

The findings of this work extend to the French population those from recent population-based studies from Germany and the United States showing, unexpectedly, lower short-term mortality among hospitalized septic patients with mental disorders compared to those without these disorders.

The study design and the analytical approach are appropriate, and the study findings are well-described. The inclusion of severity of illness scores, overweight/obesity, and type of hospital, noted as key rationale for the present vs prior studies, in risk-adjustment models strengthens the internal validity of the reported prognostic associations of mental disorders in sepsis. Notably, however, severity of illness scores, overweight/obesity, and type of hospital did not differ statistically between the groups with vs without the examined mental disorders. The authors further extend prior reports on this topic by showing that the lower mortality in septic patients with mental disorders persists over short- , intermediate- and long-term periods.

Major comments

1. The authors state in the Discussion that their findings “are paradoxically in contrast with the excess mortality due to infection in patients with SMI from previous studies [2].” However, this statement represents an erroneous comparison of studies of two different types of death rates: a study showing low *case fatality* (the present study) and one with increased *mortality rates* (the cited study and similar types of studies). The two types of death rates and the corresponding descriptive terms are not interchangeable and an illustrative discussion of their use can be found in Laupland KB, et al. Chest 2021;159:1503-06. Specifically, in the present context, comparative case fatality rates refer to death rates of patients with vs without mental disorders among those with sepsis, while mortality rates (as in reference 2) refer to death rates due to infection/sepsis among the populations at risk (e.g., the population of patients with mental disorders vs the general population).

Rather, the findings of lower case fatality among patients with mental disorders who developed septic shock in the present study are consistent with the majority of prior reports on this topic (references 13-16) in hospitalized patients with sepsis (as well as those with septic shock in some of the latter studies), while contrasting 2 other studies (references 11-12).

Notably, the premise of the present study as stated in the Abstract Background is to examine whether the reported findings of higher *mortality rates* due to infection among patients with mental disorders are similarly increased for septic shock, using the later as study hypothesis. As noted above on terminology, the usual approach to such latter study would be to conduct an investigation on the death rates due to septic shock among the population with the selected mental disorders vs in the general population. Instead, the present study design has examined case fatality among septic shock patients with vs without mental disorders. This later approach cannot answer authors’ hypothesis as stated. Similar mix occurs in authors’ justification for the present study in the last paragraph of the Introduction section, stating “To the best of our knowledge… no study accounting for the most relevant confounding variables has determined whether septic shock is associated with excess mortality in patients with SMI”. As applied to the question examined in present manuscript, the issue is rather whether SMI is associated with excess mortality among patients with septic shock (which is indeed the manuscript’s title). The Background and Conclusions of the Abstract, the relevant text of the last paragraph of the Introduction section, and the Conclusions section of the main manuscript should be revised accordingly to enhance the clarity of the study question and interpretation of the study findings.

Instead, a key inference of the findings of the present study is that the higher mortality rate due to infection/sepsis among patients with mental disorders compared to the general population appears to be due to the increased risk of infection/sepsis among the former (as noted by the authors in the Introduction) and not due to greater case fatality once they develop sepsis. This finding has major implications on future focus of resource allocation for efforts to improve life expectancy in patients with mental disorders.

Adding this latter inference to the Discussion will better clarify the study implications.

2. In the following sentence of the Discussion (referencing comment #1), the authors hypothesize on the causes of the higher mortality rates due to infection among patients with mental disorders (as in reference 2). As noted in #1 above, such postulated factors are not relevant for comparison of the present study with studies on morality rates but would be potentially applicable for comparison of the present study with others on case fatality of septic patients with vs without mental disorders.

The hypothesized greater delays in sepsis care and triage to ICU among septic patients with mental disorders vs the general population are plausible and are supported by the discussed and refenced background. However, such greater delays in care/ICU triage among septic with mental disorders were also likely present in hospitals in the present study and the authors do not provide data to suggest otherwise.

Indeed, it is likely that such postulated delays in care/ICU triage among septic patients with mental disorders were also prevalent in prior studies on this topic (references 11-16) and thus would have been expected to lead consistently to higher case fatality among the groups with mental disorders, and a similar finding would have been expected in the present study.

Instead, a key finding of the present study (and most the prior ones; references 13-16) is that some factors unique to patients with mental disorders (e.g., possibly baseline immune dysfunction, likely leading to a different, more protective, response to infection) have not only negated the adverse prognostic effects of the postulated greater delays in care/ICU triage in septic patients with mental disorders (which may have resulted in similar case fatality between the groups), but were associated with markedly lower case fatality among these patients. Such magnitude of effect estimate is remarkable and quite uncommon. No other alternative confounders, not used for modeling in the present study, appear likely to explain the study findings.

These latter issues need to be discussed as another key implication of the study findings.

3. Studies describing the prognostic implications of specific conditions or socioeconomic factors in sepsis report nearly universally findings of traits associated higher case fatality. Such findings serve as background for subsequent mechanistic studies geared to inform efforts to reduce outcome disparities.

The present study reports instead on patient groups with lower case fatality in sepsis compared to the general population. What is the clinical/research relevance of reporting such “negative” findings for sepsis care? Although the authors touch briefly and indirectly on this topic at the end of the Conclusions section, further, more explicit, discussion is warranted to address the related implications of the present study.

4. The key exposure in the present study (mental disorders) can be misclassified due to use of ICD codes (or other codes), which can affect not only specific characteristics within groups, such as obesity. Such misclassification could have affected reported effect estimates. This should be added to the study limitations.
5. The authors did not report on processes of care for sepsis, which may have differed across compared groups and thus could have led to residual confounding in modeled effects. This should be added to the study limitations.

Minor comments

1. The terms 30-day hospital morality, 90-day hospital mortality, and 1-year hospital morality should be revised by removing the word “hospital”.
2. The Methods subsection title “Procedures’ should be changed to “Exposures”.
3. The covariate termed “delay to ICU admission” (modeled as ≤1 vs > 1 day) suggests essentially lack of timely triage to ICU. However, the authors do not provide data on patients’ pre-ICU clinical status to allow such inference. The term should be changed in Tables and manuscript to time to ICU admission since hospital admission or similar term to avoid misinterpretation.
4. The Results narrative indicates that unadjusted 90-day mortality was lower among patients in all mental disorders groups. However, per Table 1, there was no statistically significant difference in 90-day mortality in between septic shock patients with vs without major depressive disorder, with similar lack of statistical significance for 30- and 1-year mortality. The text should be revised.
