# Supplementary material for: Association of severe mental illness and septic shock case fatality rate in patients admitted to the intensive care unit: A national population-based cohort study
Source: PLoS Med. 2023 Mar 13;20(3):e1004202. doi: 10.1371/journal.pmed.1004202 (PMC10042353; doi:10.1371/journal.pmed.1004202)
Supplement: Attachment — Submitted filename: ResponseLetter PLOSMED 17012023.docx [file pmed.1004202.s009.docx]

**Editor**

| **Editor’s comments** | **Authors’ response and changes made** | **Page/Line** |
| --- | --- | --- |
| 1. GENERAL  Please respond to all editor and reviewer comments detailed below, in full.  Please number the pages and lines in the manuscript starting at 1 on the abstract title and in continuous sequence thereafter.  Thank you for reporting your study according to STROBE. Please include the completed STROBE checklist as Supporting Information and refer to it in your reporting statement in the methods section. When completing the checklist, please use section and paragraph numbers, rather than page or line numbers.  Please consider how you frame your research question and the terminology you use when describing it – see comments below from the academic editor and reviewers.  The title, parts of the abstract, introduction and discussion imply that this is a study of whether people with serious mental illness, have lower mortality from sepsis. However, both the reviewers and the academic editor have raised concerns regarding this inference for multiple reasons, which the editorial team are in agreement with. Please see their detailed comments below and revise accordingly. | We provided responses to the reviewers’ insightful comments and explain how we incorporated their comments into the revised manuscript  The pages and lines are now indicated in the manuscript.  The STROBE CHECKLIST has been completed and is included as Supporting Information and referred to in the text.  In accordance with the comments of the Editor and Reviewer 1, we have corrected this inference throughout the manuscript, changing mortality to case fatality. | Not applicable  Not applicable  Not applicable  Not applicable |
| COMMENTS FROM THE ACADEMIC EDITOR  I agree with the decision about major revision, but I have the following additional comments.  1. Building on the comments from reviewer 1, there does seem to be a confusion around the research question being addressed and, as a consequence, the interpretation of the findings. I understand the research question to effectively be 'after adjusting for all the other factors (social, physical health, habits) that make people with SMI at risk of excess mortality, is there something about SMI as a disease process/its pharmacological treatment that might affect risk of mortality in those who have sepsis and who manage to access ICU ?'. This is not a study of whether people with SMI as a group have lower mortality from sepsis as implied by the title, the abstract conclusion and in other places in the paper. As the authors start to raise in the discussion, it is very likely that we are seeing a survival bias in the sample being examined - this is not the full sample of people with SMI and sepsis in the population. It does not include people with SMI and sepsis who had sepsis and died in the community, in the hospital before ICU access or, indeed, in the first 48 hours of ICU care. The actual research question being addressed has merit, but it should not be conflated with a research question about mortality from sepsis in people with SMI in general. For the background, I note that excess mortality in people with SMI is strongly related to social disadvantage - please add that to this list alongside unhealthy behaviours, increased infection risk, etc. | We thank the Editor and the Reviewer 1 for this major comment that we have considered in the whole manuscript. Our findings concern case fatality rates in patients with septic shock admitted to the intensive care unit.  First, we changed the title from: “Severe mental illness is associated with a better prognosis in septic shock: results from 187,587 hospitalizations in France”  to (after consideration of the PLOS medicine guidelines): “Association of severe mental illness and septic shock case fatality rate in patients admitted to the intensive care unit: a national population-based cohort study”.  Second, we corrected the objective and the conclusion of the abstract as follows  “The primary objective was to compare the 90-day CFR in septic shock patients with and without SMI admitted to the intensive care unit (ICU)”.  “~~In contrast to the excess mortality from infection observed in patients with SMI, our findings suggested improved outcome from septic shock in the ICU.~~ Our findings suggest improved septic shock outcome in patients with SMI compared to patients without. This finding may be the result of different immunological profiles and exposures to psychotropic medications, which should be further explored.”  Third, we corrected the objective at the end of the introduction as follows:  “The primary objective was to compare 90-day CFRs in septic shock patients with and without SMI admitted to the intensive care unit (ICU).”  Last, we agree with the Editor and have now added the social disadvantage of patients with SMI to the background: “This is mainly attributed to higher rates of physical disease, social disadvantage, unhealthy lifestyle behaviors, and inadequate health care in SMI patients compared to in the general population [3–6].” | P1/L1  P2/L45  P7/L140  P6/L109 |
| 2. Please give further explanation for the exclusion of people with a diagnosis of sepsis in the first 48 hours of ICU admission. | As explained in the manuscript, we limited inclusion to patients with an ICU length of stay of at least 48 h, unless the patient died within 48 h, in order to avoid over-estimating diagnoses of septic shock.  Although the coding of septic shock has been strictly regulated since the DRG system was introduced in France, we cannot exclude over-coding due to the high tariff associated with the codes, especially for short stays in the ICU. Indeed, the length of stay for patients with septic shock is about 7 days (IQR 3-14 days) [Sakr, 2018]. In addition, this estimation included deceased and non-deceased patients. We therefore considered Q1 (<=2 days) to be a credible threshold below which the probability of having septic shock was low (excluding, patients who died within these 48 hours).  Sakr et al. Sepsis in Intensive Care Unit Patients: Worldwide Data From the Intensive Care over Nations Audit. Open Forum Infect Dis. 2018 Nov 19;5(12):ofy313.  We have added this explanation in the methods section of the manuscript. | P7/L168 |
| 3. Another reviewer raised the need to look in more detail into the potential reasons for the difference in mortality that might not be due to immunological profiles/medicines. There are some differences, not all significant but nonetheless present - lower malignancies and lower co-morbid conditions. Although these are adjusted for, it is possible that there is residual confounding. The SES indicators is also area level and may lead to residual confounding (people with SMI more likely to be from private hospitals indicates that there could actually be SES differences that are inadequately measured). | As suggested by the Editor and Reviewer 3, we added the following explanation: “However, there were some important differences between patients with and without SMI after matching (e.g., fewer malignancies and fewer comorbid conditions), which may have influenced outcomes. Although these differences were adjusted for, it is possible that residual confounding remained. In addition, the social deprivation indicator is based on area-level and may thus also lead to residual confounding.” | P14/L363 |
| The level of 'other substance addiction' seems to be very high (in both people with SMI and those without). I might have missed it, but can you add the main substances that were contributing to this (if you have the information). | We apologize for this error, the substance addiction presented in the table was the “total substance addiction” (including tobacco and alcohol) and not “other substance addiction”. We have now corrected the number and percentage, which are lower. We have also present the main substances: opioid-related disorders, cannabis-related disorders, cocaine-related disorders, and other substances. | P27 |
| TITLE  Please revise your title in context of the academic editor and reviewer comments and according to PLOS Medicine's style. Your title must be nondeclarative and not a question. It should begin with the main concept if possible. "Effect of" should be used only if causality can be inferred, i.e., for an RCT. Please place the study design ("A randomized controlled trial," "A retrospective study," "A modelling study," etc.) in the subtitle (ie, after a colon). | We changed the title in accordance with the academic editor and reviewer comments and according to PLOS Medicine's style as follows:  “Severe mental illness is associated with a better prognosis in septic shock: results from 187,587 hospitalizations in France”  to  “Association of severe mental illness and septic shock case fatality rate in patients admitted to the intensive care unit: a national population-based cohort study” | P1/L1 |
| DATA AVAILABILITY STATEMENT  Thank you for including a Data Availability Statement (DAS) which requires revision. For each data source used in your study:  a) If the data are freely or publicly available, note this and state the location of the data: within the paper, in Supporting Information files, or in a public repository (include the DOI or accession number).  b) If the data are owned by a third party but freely available upon request, please note this and state the owner of the data set and contact information for data requests (web or email address). Note that a study author cannot be the contact person for the data.  c) If the data are not freely available, please describe briefly the ethical, legal, or contractual restriction that prevents you from sharing it. Please also include an appropriate contact (web or email address) for inquiries (again, this cannot be a study author). | The data are not freely available, they are accessible on a French national platform (ATIH). No personal data can be extracted from this platform (even for those who have access), only aggregated results can be extracted. All the information can be asked at this address: <https://www.atih.sante.fr/nous-contacter>. | Not applicable |
| ABSTRACT  Abstract Background:  Please see academic editor comments above and reviewer comments below, which we agree with, and revise the abstract background accordingly. Provide the context of why the study is important. The final sentence should clearly state the study question. | The abstract background has been changed as follows:  “Patients with severe mental illness (SMI) (*i.e.*, schizophrenia, bipolar disorder, or major depressive disorder) have been reported to have excess mortality rates from infection compared to patients without SMI~~. We hypothesized the same would be true for septic shock,~~ but whether SMI is associated with higher or lower case fatality rates (CFRs) associated with infection remains unclear. The primary objective was to compare the 90-day CFR in septic shock patients with and without SMI admitted to the intensive care unit (ICU).” | P3/L40 |
| Abstract Methods and Findings:  “90-day hospital mortality” – did all these patients die in hospital? If not which we suspect is the case then please report “90-day mortality”. See below (methods and results) also, please check and clarify or amend throughout as necessary. | We have worked on the French National Hospital database, all the patients died in hospital (same stay or rehospitalisation).  In France, the majority of patients die in hospital. In our study, the CFRs at 30 and 90 days were similar to those reported in other works. In addition, the evolution of the CFR between 30 and 90 days and between 90 and 365 days is similar between the patients with and without SMI, supporting a lack of bias to account for the different extra-hospital mortality.  We propose adding the following limitation in the discussion:  “First, we described only patients who died in hospital, which means that the CFR might be slightly underestimated. However, deaths occurring outside the hospital are extremely rare in France [28]. Our findings at 30 and 90 days were similar to those reported in other studies [69]. In addition, the evolution of the CFR between 30 and 90 days and between 90 and 365 days was similar in the patients with and without SMI, supporting a lack of bias to account for the different extra-hospital mortality.”  28. Armoiry X, Obadia J-F, Pascal L, Polazzi S, Duclos A. Comparison of transcatheter versus surgical aortic valve implantation in high-risk patients: A nationwide study in France. J Thorac Cardiovasc Surg. 2018;156: 1017-1025.e4. doi:10.1016/j.jtcvs.2018.02.092  69. Bauer M et al. Mortality in sepsis and septic shock in Europe, North America and Australia between 2009 and 2019- results from a systematic review and meta-analysis Crit Care. 2020 May 19;24(1):239. | P14/L381 |
| Statistical reporting is a bit confusing here:  -It is unclear whether the results you present are adjusted or unadjusted (see statistical reviewer comments also) if you mention both would it be reasonable to report both?  -Detailing the number of controls within each group as you have done distracts from the data reporting and because this information is placed next to percentages it is easy to think these numbers contribute to deriving the percentages, but they do not. Please revise.  -Numerators and denominators used to derive percentages should be clearly reported  -Where 95% CIs are reported, please also report p-values  -Suggest reporting statistical information as follows to improve accessibility to the reader: “HR 0.83, 95%CI [0.79, 0.88], p =/<” commas separating upper and lower confidence limits may help to mitigate against any confusion regarding negative values. Where p-values are significant please report as less than the significance level (<0.01 or <0.05 an so on) | We apologize for this imprecision. To address this point, we suggest removing “before and after adjustment” which is not necessary and just present the main results of the manuscript: adjusted analyses with adjusted HR.  For a better readability, we have removed the number of controls.  We have also removed the percentages in the first sentence because they do not add important information: “A total of 187,587 adult patients with septic shock admitted to the ICU were identified, including 3,812 with schizophrenia ~~(2.0%)~~, 2,258 with bipolar disorder ~~(1.2%)~~, and 5,246 with major depressive disorder ~~(2.8%)~~. Compared to matched controls, ~~before and after adjustment,~~ the 90-day CFR was significantly lower in patients with schizophrenia (1,052/3,269=32.2% vs. 5,000/10,894=45.5% ~~(controls: n=10,894)~~; adjusted hazard ratio (aHR)=0.70, 95% confidence interval (CI) 0.65,0.75, p<0.001), bipolar disorder (632/1,923=32.9% vs. 2,854/6,303=45.3% ~~(controls: n=6,303)~~; aHR=0.70, 95%CI=0.63,0.76, p<0.001), and major depressive disorder (1,834/4,432=41.4% vs. 6,798/14,452=47.1% ~~(controls: n=14,452)~~; aHR=0.85, 95%CI=0.81,0.90, p<0.001).”  We followed this recommendation, see the text above.  We followed this recommendation, see the text above. | P2/L59 |
| In the last sentence of the Abstract Methods and Findings section, please describe the main limitation(s) of the study's methodology | We have added the following limitations: “Study limitations include inability to capture deaths occurring outside hospital, lack of data on processes of care, and problems associated with missing data and miscoding in medico-administrative databases.” | P3/L68 |
| Abstract conclusions:  Please see above and revise in-line with the reviewer and academic editor comments below.  Please address the study implications without overreaching what can be concluded from the data and interpret the study based on the results presented in the abstract, emphasizing what is new without overstating your conclusions.  Please avoid vague statements such as "these results have major implications for policy/clinical care". Mention only specific implications substantiated by the results.  Please avoid assertions of primacy ("We report for the first time....") | We revised the conclusion of the abstract as follows  “~~In contrast to the excess mortality from infection observed in patients with SMI, our findings suggested improved outcome from septic shock in the ICU.~~ Our findings suggest improved septic shock outcome in patients with SMI compared to patients without. This finding may be the result of different immunological profiles and exposures to psychotropic medications, which should be further explored.” | P4/L71 |
| AUTHOR SUMMARY  At this stage, we ask that you include a short, non-technical Author Summary of your research to make findings accessible to a wide audience that includes both scientists and non-scientists. The Author Summary should immediately follow the Abstract in your revised manuscript. This text is subject to editorial change and should be distinct from the scientific abstract. Please see our author guidelines for more information: https://urldefense.com/v3/__https://journals.plos.org/plosmedicine/s/revising-your-manuscript*loc-author-summary__;Iw!!JQ5agg!cT4ulSOlUtVq_8mNTimWwS0rnR1hQOqaZpSCwqbcqV3pllylCEmMB0wpjaK8piaHPs2Mvm9XjbZtJ0XbVQeNdw$ | *Why was this study done?*   - Patients with severe mental illness (SMI) have been reported to have excess mortality from sepsis (number of deaths due to sepsis in the whole population). - Whether SMI is associated with higher or lower sepsis-associated case fatality remains unclear (number of deaths due to sepsis in the population with sepsis). - No study has determined whether SMI is associated with excess case fatality in patients with septic shock, the most severe form of sepsis when accounting for the most relevant confounding variables.   *What did the researchers do and find?*   - In this nationwide, population-based cohort study, we compared 30-, 90- and 365-day case fatality rates (CFR) in septic shock patients with and without SMI admitted to the intensive care unit (ICU). - We identified 187,587 adult patients with septic shock admitted to the ICU, including 3,812 with schizophrenia, 2,258 with bipolar disorder, and 5,246 with major depressive disorder. - The 30-, 90- and 365-day CFRs were lower in patients with SMI than in patients without SMI after controlling for multiple potential confounding factors (using intrahospital matching and adjustments for multiple comorbidities and illness severity) and addressing potential biases not considered in previous studies.   *What do these findings mean?*   - Our findings suggest improved septic shock outcomes in patients with SMI compared to patients without. - This finding may be the result of different immunological profiles and exposures to psychotropic medications, a hypothesis that needs to be confirmed in future studies. | P5/L79 |
| INTRODUCTION  Please revise your introduction in mind of the academic editor and reviewer comments  Please ensure you have addressed past research and explain the need for and potential importance of your study. Indicate whether your study is novel and how you determined that. If there has been a systematic review of the evidence related to your study (or you have conducted one), please refer to and reference that review and indicate whether it supports the need for your study.  Please conclude the Introduction with a clear description of the study question or hypothesis. | The introduction has been corrected according to the Editor and Reviewer comments, especially related to the difference between mortality and case-fatality that had to be better articulated. Past research reported higher mortality in patients with SMI from infection but whether SMI is associated with increased or decreased case-fatality from infection (i.e., the proportion of persons with infection who die from that infection) compared with the general population is unclear. To date, no study accounting for the most relevant confounding variables has determined whether SMI is associated with excess case-fatality in patients with septic shock. We hypothesized that patients with SMI had an increased case-fatality rate in comparison with patients without SMI.  The introduction has modified as follows:  “Data have consistently indicated that individuals with severe mental illness (SMI) (*i.e.,* schizophrenia, bipolar disorder, or major depressive disorder) are at higher risk of premature mortality than the general population [1,2]. This is mainly attributed to higher rates of physical disease, social disadvantage, unhealthy lifestyle behaviors, and inadequate health care in patients with SMI ~~compared to in the general population~~ [3–6]. Among somatic diseases, infections are disproportionately more frequent in patients with SMI than in the general population, representing a potentially avoidable contributor to early death [2,7,8]. In a meta-analysis, patients with SMI were reported to have higher mortality rates from infection than the general population~~, but the findings showed substantial heterogeneity and sepsis was not specifically studied~~ [2].  Whether SMI is associated with higher or lower infection-associated case fatality (*i.e.*, the proportion of persons with infection who die from that infection [9]) compared with the general population is unclear.  (…)  We hypothesized that patients with SMI would have a higher septic shock CFR than patients without SMI.” | P6/L109  P6/L113  P6/116  P7/L144 |
| METHODS and RESULTS  Under paragraph titled “outcomes” please elaborate on what you mean by “clinical characteristics”  Under paragraph titled “collected data” suggest “(…substance use)”  What does “origin of the patient” mean? Please clarify perhaps with the use of an alternative term | We have replaced “clinical characteristics” by “clinical profiles of patients ».  We have replaced “origin of the patient” by “source of hospital admission (*i.e.*, where the patient came from [home, transfer from other hospital ward])”. | P8/L178  P8/L192 |
| Where 95% CIs are reported, please also report p-values and the statistical tests used to determine them. | As suggested, we have added the p-values in the text and the tables. | Not applicable |
| As in the abstract, suggest reporting statistical information as follows to improve accessibility to the reader: “HR 0.83, 95%CI [0.79, 0.88], p =/<” commas separating upper and lower confidence limits may help to mitigate against any confusion regarding negative values. Where p-values are significant please report as less than the significance level (<0.01 or <0.05 for example). | We followed this recommendation. | P3 |
| Under paragraph titled “comparison of mortality rates”: You state: “similar findings for 30-, 90- and 365-day hospital mortality” do you report hospital mortality or were some patients discharged? Please clarify and as necessary amend removing the word “hospital” so as to report “30-, 90-, 365-day mortality”. Please check carefully and revise throughout, including in the abstract. | We worked on the French National Hospital database, so all the patients died in hospital (same stay or rehospitalisation).  In France, the majority of patients die in hospital. In our study, the CFRs at 30 and 90 days were similar to those reported in other works. In addition, the evolution of CFR between 30 and 90 days and between 90 and 365 days was similar patients with and without SMI, supporting a lack of bias to account for the different extra-hospital mortality rate.  We propose adding the following limitation in the discussion:  “First, we described only patients who died in hospital, which means that the CFR might be slightly underestimated. However, deaths occurring outside the hospital are extremely rare in France [28]. Our findings at 30 and 90 days were similar to those reported in other studies [69]. In addition, the evolution of the CFR between 30 and 90 days and between 90 and 365 days was similar in the patients with and without SMI, supporting a lack of bias to account for the different extra-hospital mortality.”  28. Armoiry X, Obadia J-F, Pascal L, Polazzi S, Duclos A. Comparison of transcatheter versus surgical aortic valve implantation in high-risk patients: A nationwide study in France. J Thorac Cardiovasc Surg. 2018;156: 1017-1025.e4. doi:10.1016/j.jtcvs.2018.02.092  69. Bauer M et al. Mortality in sepsis and septic shock in Europe, North America and Australia between 2009 and 2019- results from a systematic review and meta-analysis Crit Care. 2020 May 19;24(1):239. | P14/L381 |
| TABLES  Please provide a table showing the baseline characteristics of the study population in the main manuscript as table 1.  Tables 1 and 2: Please quantify the results with p-values and 95% CI. If there is a specific reason (s) why it would be preferable to report standardized difference, please clearly state those reasons. | The tables are now completed.  Regarding the SD, we have now added p-values and 95% CI.  In a general way, the p-value can inform the reader whether an effect exists but the p-value will not reveal the size of the effect. Effect size helps readers understand the magnitude of differences found.  In our study, we have large sample sizes which make it difficult to interpret the p-value. Indeed, the statistical significance depends upon both sample size and effect size. For this reason, p-values are considered to be confounded because of their dependence on sample size. Sometimes a statistically significant result means only that a large sample size was used with quite small and possibly trivial differences.  Gail M Sullivan, Richard Feinn. Using Effect Size-or Why the P Value Is Not Enough.  J Grad Med Educ. 2012 Sep;4(3):279-82.  We have therefore revised the method section of the manuscript as follows:  “Standardized differences were used to compare patients with and without SMI using weights to normalize the distribution of patients. An absolute standardized difference (SD) of ⩽0.20 was chosen to indicate a negligible difference in the mean or prevalence of a variable between groups [33]. The SD helps to understand the magnitude of the differences found, in addition to statistical significance which examines whether the findings are likely to be due to chance [Sullivan, 2012].” | P9/L216 |
| FIGURES  Please see reviewer 2’s (statistical reviewer) comments regarding the presentation of the models  Figure 1: Please define ICU, we suggest in an expanded figure title  Figure 2: Thank you for including unadjusted analyses. Please remove the word “hospital” from the title. Please indicate the meaning of the dots and lines in the figure caption  Please address the same in the supporting figures | As suggested by Reviewer 2, we have now presented one main adjusted model. All the sensitivity analyses are now in the supplementary figures.  We have now added and defined ICU in the title.  We have moved this figure to the Supplement. We have left the word “hospital” in the title but explain better in the manuscript that we described only patients who died in hospitals (abstract, method, discussion).  We have indicated the meaning of dots (square) and lines. | P27  P40 |
| DISCUSSION  Please see reviewer 1 comments below regarding interpretation/implication of your study findings of your data.  Please expand the reporting of limitations in-line with reviewer comments  Please remove the sub-heading “conclusions” such that the discussion reads as a single piece of continuous prose.  Please remove the conflict of interest statement from the main manuscript and place only in the submission form when you re-submit your manuscript. | The discussion has been corrected according to the comments of the reviewers (1 and 3), with a more balanced interpretation/implication. We now have three main implications: 1. The reasons for the differences in survival between patients with SMI and controls could not be determined in our study, but may include differences in immunological profiles and exposures to the immunomodulation effect of medications; 2. A potential implication of these results is that the frequently observed discontinuation of psychotropic medications on admission to the ICU should be carefully considered given the risks of relapse of the psychiatric disorder and their potential benefits of these drugs on mortality in the context of septic shock; 3. The lower case-fatality rate may have health policy implications on future focus of resource allocation to improve life expectancy in patients with SMI. This finding suggests that the higher mortality rate due to infection/sepsis among patients with SMI reported in previous studies appears to be due to the increased risk of infection/sepsis among patients with SMI and not due to greater case-fatality once they have developed sepsis.  However, in accordance with Reviewer 2 and the Editor, we added that there were some important differences between patients with and without SMI after matching (e.g., fewer malignancies and fewer comorbid conditions, which may have influenced outcomes. Although these are adjusted for, it is possible that there is residual confounding. In addition, the social deprivation indicator is area level and may thus also lead to residual confounding.  We have expanded the limitation section with 3 additional limitations: possible bias with in-hospital mortality, absence of data on processes of care and problem of missing data and miscoding: “Our study has several limitations. First, we described only patients who died in hospital, which means that the CFR might be slightly underestimated. However, deaths occurring outside the hospital are extremely rare in France [28]. Our findings at 30 and 90 days were similar to those reported in other studies [69]. In addition, the evolution of the CFR between 30 and 90 days and between 90 and 365 days was similar in the patients with and without SMI, supporting a lack of bias to account for the different extra-hospital mortality. Second, a weakness of administrative databases is the potential miscoding of diagnoses during hospital stays, which can underestimate important patient features (especially for overweight and obesity which are insufficiently coded in administrative databases but which allow the most serious cases to be targeted for epidemiological research [70,71]) and disease severity at ICU admission. Missing data are thus assumed to indicate no disease present. In addition, the key exposure in the present study (*i.e.*, SMI) can be misclassified due to use of ICD-10 codes, which could have affected reported effect estimates. However, the coding has been strictly regulated since the DRG system was introduced in France. To control for these weaknesses, we used a matching procedure and adjustment based on a large number of patient characteristics and controlling for confounders at the hospital level. The matching process failed for 15% of patients due to the age imbalance between patients with and without SMI. However, the sensitivity analysis on the whole cohort reported similar findings. There are also limitations associated with the lack of some variables, including specific description of psychotropic medications, body mass index, fitness, blood lactate levels, which could be useful to categorize our patients. Furthermore, the time between the onset of infection and the need for vasopressor support could not be determined. Some patients may require vasopressor support for a problem other than septic shock. Finally, processes of care for sepsis were not analyzed in detail in our study and may have differed across compared groups, which could have led to residual confounding in modelled effects.”  We have removed the sub-heading “conclusions”.  We have removed the COI from the manuscript. | P14/L381 |
| SUPPORTING INFORMATION  See above (figures)  Supplementary figure 3: please define ICU in the caption/title  Supplementary tables 1, 3, 4: Please quantify the results with p-values and 95% CI. If there is a specific reason (s) why it would be preferable to report standardized difference, please clearly state those reasons. | ICU is now defined in the title  We agree with the Editor and Reviewer 2, we have now added p-values and 95% CI when necessary.  In a general way, the p-value can inform the reader whether an effect exists but the p-value will not reveal the size of the effect. Effect size helps readers understand the magnitude of the differences found.  In our study, we have large sample sizes which make it difficult to interpret the p-value. Indeed, the statistical significance depends upon both sample size and effect size. For this reason, p-values are considered to be confounded because of their dependence on sample size. Sometimes a statistically significant result means only that a large sample size was used with quite small and possibly trivial differences.  Gail M Sullivan, Richard Feinn. Using Effect Size-or Why the P Value Is Not Enough.  J Grad Med Educ. 2012 Sep;4(3):279-82.  We propose completing the method section of the manuscript as follows:  “Standardized differences were used to compare patients with and without SMI using weights to normalize the distribution of patients. An absolute standardized difference (SD) of ⩽0.20 was chosen to indicate a negligible difference in the mean or prevalence of a variable between groups [33]. The SD helps to understand the magnitude of the differences found, in addition to statistical significance which examines whether the findings are likely to be due to chance [Sullivan, 2012].” | P40  Not applicable  P9/L216 |

**Reviewer 1**

| **Reviewer’s comments** | **Authors’ response and changes made** | **Page** |
| --- | --- | --- |
| 1. The manuscript describes the association of three categories of mental disorders with mortality among ICU-managed patients with septic shock in a population-based cohort.  The findings of this work extend to the French population those from recent population-based studies from Germany and the United States showing, unexpectedly, lower short-term mortality among hospitalized septic patients with mental disorders compared to those without these disorders.  The study design and the analytical approach are appropriate, and the study findings are well-described. The inclusion of severity of illness scores, overweight/obesity, and type of hospital, noted as key rationale for the present vs prior studies, in risk-adjustment models strengthens the internal validity of the reported prognostic associations of mental disorders in sepsis. Notably, however, severity of illness scores, overweight/obesity, and type of hospital did not differ statistically between the groups with vs without the examined mental disorders. The authors further extend prior reports on this topic by showing that the lower mortality in septic patients with mental disorders persists over short- , intermediate- and long-term periods. | We thank Reviewer 1 for her/his careful reading and comments that helped us to improve our work. | Not applicable |
| 2. The authors state in the Discussion that their findings "are paradoxically in contrast with the excess mortality due to infection in patients with SMI from previous studies [2]." However, this statement represents an erroneous comparison of studies of two different types of death rates: a study showing low case fatality (the present study) and one with increased mortality rates (the cited study and similar types of studies). The two types of death rates and the corresponding descriptive terms are not interchangeable and an illustrative discussion of their use can be found in Laupland KB, et al. Chest 2021;159:1503-06. Specifically, in the present context, comparative case fatality rates refer to death rates of patients with vs without mental disorders among those with sepsis, while mortality rates (as in reference 2) refer to death rates due to infection/sepsis among the populations at risk (e.g., the population of patients with mental disorders vs the general population)  Rather, the findings of lower case fatality among patients with mental disorders who developed septic shock in the present study are consistent with the majority of prior reports on this topic (references 13-16) in hospitalized patients with sepsis (as well as those with septic shock in some of the latter studies), while contrasting 2 other studies (references 11-12).  Notably, the premise of the present study as stated in the Abstract Background is to examine whether the reported findings of higher mortality rates due to infection among patients with mental disorders are similarly increased for septic shock, using the later as study hypothesis. As noted above on terminology, the usual approach to such latter study would be to conduct an investigation on the death rates due to septic shock among the population with the selected mental disorders vs in the general population. Instead, the present study design has examined case fatality among septic shock patients with vs without mental disorders. This later approach cannot answer authors' hypothesis as stated. Similar mix occurs in authors' justification for the present study in the last paragraph of the Introduction section, stating "To the best of our knowledge… no study accounting for the most relevant confounding variables has determined whether septic shock is associated with excess mortality in patients with SMI". As applied to the question examined in present manuscript, the issue is rather whether SMI is associated with excess mortality among patients with septic shock (which is indeed the manuscript's title). The Background and Conclusions of the Abstract, the relevant text of the last paragraph of the Introduction section, and the Conclusions section of the main manuscript should be revised accordingly to enhance the clarity of the study question and interpretation of the study findings.  Instead, a key inference of the findings of the present study is that the higher mortality rate due to infection/sepsis among patients with mental disorders compared to the general population appears to be due to the increased risk of infection/sepsis among the former (as noted by the authors in the Introduction) and not due to greater case fatality once they develop sepsis. This finding has major implications on future focus of resource allocation for efforts to improve life expectancy in patients with mental disorders.  Adding this latter inference to the Discussion will better clarify the study implications. | We have now clarified throughout the whole manuscript by replacing mortality by case-fatality: title, abstract…  The new title is: “Association of severe mental illness and septic shock case fatality rate in patients admitted to the intensive care unit: a national population-based cohort study”  In addition, we removed this section of the discussion which was too speculative: “~~Our findings also question the health care system responsiveness in patients with SMI and more specifically the access to ICU. Indeed, our findings are paradoxically in contrast with the excess mortality due to infection observed in patients with SMI from previous studies [2]. One hypothesis is that this excess mortality found in previous works is due to delays in the management of the SMI patients (because of late diagnosis) and lack of access to ICU. Compared with the general population, previous studies have shown that patients with SMI have longer delays in somatic diagnoses and less frequently received appropriate care, as a result of physician-related (e.g., stigma, diagnostic overshadowing) and patient-related (e.g., poor insight into potential symptoms, reduced likelihood to seek help) factors [5,6,51–53]. A typical example of sepsis under-diagnosis in a patient with SMI would be a confused and hypoxemic patient [54], in whom the diagnosis of infection could be delayed due to alternative clinical hypotheses including oversedation or drug interactions [55]. These findings could also raise the suggestion of deliberate triage at ICU admission for patients with SMI, as proposed in a recent study [25]. Future work should thus explore the mortality and trajectory of patients with SMI upstream of their admission to ICU to confirm our hypothesis.~~”  This section was replaced by the following one in accordance with the interpretation of the Reviewer: “A key finding of our study (and most of the prior ones [14–17]) is that some factors unique to patients with SMI (*e.g.*, possibly baseline immune dysfunction leading to a different, more protective, response to infection) not only negated the adverse prognostic effects of SMI in septic shock patients (which could have resulted in similar case fatality between the groups), but were associated with markedly lower case fatality among these patients. The magnitude of this effect estimate is remarkable, especially in this vulnerable population marked by low socioeconomic status. However, there were some important differences between patients with and without SMI after matching (*e.g.*, fewer malignancies and fewer comorbid conditions), which may have influenced outcomes. Although these differences were adjusted for, it is possible that residual confounding remained. In addition, the social deprivation indicator is based on area-level and may thus also lead to residual confounding.” | P1/L1  P13/L357 |
| 3. In the following sentence of the Discussion (referencing comment #1), the authors hypothesize on the causes of the higher mortality rates due to infection among patients with mental disorders (as in reference 2). As noted in #1 above, such postulated factors are not relevant for comparison of the present study with studies on morality rates but would be potentially applicable for comparison of the present study with others on case fatality of septic patients with vs without mental disorders.  The hypothesized greater delays in sepsis care and triage to ICU among septic patients with mental disorders vs the general population are plausible and are supported by the discussed and refenced background. However, such greater delays in care/ICU triage among septic with mental disorders were also likely present in hospitals in the present study and the authors do not provide data to suggest otherwise.  Indeed, it is likely that such postulated delays in care/ICU triage among septic patients with mental disorders were also prevalent in prior studies on this topic (references 11-16) and thus would have been expected to lead consistently to higher case fatality among the groups with mental disorders, and a similar finding would have been expected in the present study.  Instead, a key finding of the present study (and most the prior ones; references 13-16) is that some factors unique to patients with mental disorders (e.g., possibly baseline immune dysfunction, likely leading to a different, more protective, response to infection) have not only negated the adverse prognostic effects of the postulated greater delays in care/ICU triage in septic patients with mental disorders (which may have resulted in similar case fatality between the groups), but were associated with markedly lower case fatality among these patients. Such magnitude of effect estimate is remarkable and quite uncommon. No other alternative confounders, not used for modeling in the present study, appear likely to explain the study findings.  These latter issues need to be discussed as another key implication of the study findings. | As previously explained and in accordance with the comments of the Reviewer and Editor, this section on delays in care/triage… was too speculative and was removed. However, we appreciated the following interpretation that is now added in the discussion as follows:  “A key finding of our study (and most of the prior ones [14–17]) is that some factors unique to patients with SMI (*e.g.*, possibly baseline immune dysfunction leading to a different, more protective, response to infection) not only negated the adverse prognostic effects of SMI in septic shock patients (which could have resulted in similar case fatality between the groups), but were associated with markedly lower case fatality among these patients. The magnitude of this effect estimate is remarkable, especially in this vulnerable population marked by low socioeconomic status.” | P13/L357 |
| 4. Studies describing the prognostic implications of specific conditions or socioeconomic factors in sepsis report nearly universally findings of traits associated higher case fatality. Such findings serve as background for subsequent mechanistic studies geared to inform efforts to reduce outcome disparities.  The present study reports instead on patient groups with lower case fatality in sepsis compared to the general population. What is the clinical/research relevance of reporting such "negative" findings for sepsis care? Although the authors touch briefly and indirectly on this topic at the end of the Conclusions section, further, more explicit, discussion is warranted to address the related implications of the present study. | This finding is difficult to explain. As suggested by the Editor, we have added the following nuance after the sentence on the socioeconomical status: “However, there were some important differences between patients with and without SMI after matching (*e.g.*, fewer malignancies and fewer comorbid conditions), which may have influenced outcomes. Although these differences were adjusted for, it is possible that residual confounding remained. In addition, the social deprivation indicator is based on area-level and may thus also lead to residual confounding.”  In addition, as suggested by the Reviewer 3, we propose the following implication at the end of the first part of the discussion: “A potential implication of these results is that the frequently observed discontinuation of psychotropic medications on admission to the ICU should be carefully considered given the risks of relapse of the psychiatric disorder as well as the potential benefits of these drugs on mortality in the context of septic shock.” | P14/L363  P13/L335 |
| 5. The key exposure in the present study (mental disorders) can be misclassified due to use of ICD codes (or other codes), which can affect not only specific characteristics within groups, such as obesity. Such misclassification could have affected reported effect estimates. This should be added to the study limitations. | We agree with the Reviewer. We have added this limitation as follows:  “Second, a weakness of administrative databases is the potential miscoding of diagnoses during hospital stays, which can underestimate important patient features (especially for overweight and obesity which are insufficiently coded in administrative databases but which allow the most serious cases to be targeted for epidemiological research [70,71]) and disease severity at ICU admission. Missing data are thus assumed to indicate no disease present. In addition, the key exposure in the present study (*i.e.*, SMI) can be misclassified due to use of ICD-10 codes, which could have affected reported effect estimates.” | P14/L387 |
| 6. The authors did not report on processes of care for sepsis, which may have differed across compared groups and thus could have led to residual confounding in modeled effects. This should be added to the study limitations. | We agree with the Reviewer. We have added this limitation as follows: “Finally, processes of care for sepsis were not analyzed in detail in our study and may have differed across compared groups, which could have led to residual confounding in modelled effects.” | P15/L404 |
| 7. The terms 30-day hospital morality, 90-day hospital mortality, and 1-year hospital morality should be revised by removing the word "hospital". | We worked on the French National Hospital database, so all the patients died in hospital (same stay or rehospitalisation).  In France, the majority of patients die in hospital. In our study, the CFR at 30 and 90 days are similar with those reported in other works. In addition, the evolution of CFR between 30 and 90 days and between 90 and 365 days is similar in patients with and without SMI, supporting a lack of bias to account for the different extra-hospital mortality rate.  We propose adding the following limitation in the discussion:  “First, we described only patients who died in hospital, which means that the CFR might be slightly underestimated. However, deaths occurring outside the hospital are extremely rare in France [28]. Our findings at 30 and 90 days were similar to those reported in other studies [69]. In addition, the evolution of the CFR between 30 and 90 days and between 90 and 365 days was similar in the patients with and without SMI, supporting a lack of bias to account for the different extra-hospital mortality.”  28. Armoiry X, Obadia J-F, Pascal L, Polazzi S, Duclos A. Comparison of transcatheter versus surgical aortic valve implantation in high-risk patients: A nationwide study in France. J Thorac Cardiovasc Surg. 2018;156: 1017-1025.e4. doi:10.1016/j.jtcvs.2018.02.092  69. Bauer M et al. Mortality in sepsis and septic shock in Europe, North America and Australia between 2009 and 2019- results from a systematic review and meta-analysis Crit Care. 2020 May 19;24(1):239. | P14/L381 |
| 8. The Methods subsection title "Procedures' should be changed to "Exposures". | As suggested by the Reviewer, we have replaced “Procedures” par “Exposures”. | P9/L196 |
| 9. The covariate termed "delay to ICU admission" (modeled as ≤1 vs > 1 day) suggests essentially lack of timely triage to ICU. However, the authors do not provide data on patients' pre-ICU clinical status to allow such inference. The term should be changed in Tables and manuscript to time to ICU admission since hospital admission or similar term to avoid misinterpretation. | As suggested by the Reviewer, we have replaced “delay to ICU admission” par “the length of time between hospital admission and ICU admission”. | P8/L193 |
| 10. The Results narrative indicates that unadjusted 90-day mortality was lower among patients in all mental disorders groups. However, per Table 1, there was no statistically significant difference in 90-day mortality in between septic shock patients with vs without major depressive disorder, with similar lack of statistical significance for 30- and 1-year mortality. The text should be revised. | As suggested by the Editor and Reviewer 2, we have completed our analyses (standardized difference) with statistical tests. The p-values were significant for the three SMI groups. | Not applicable |

**Reviewer 2**

| **Reviewer’s comments** | **Authors’ response and changes made** | **Page/Line** |
| --- | --- | --- |
| 1. This is an interesting study on the association between severe mental illness and 90-day hospital mortality in septic shock patients in France. However, there are quite a few major issues needing attention. | We appreciate the careful reading of Reviewer 2 that gave us the opportunity to improve the quality of the manuscript. | Not applicable |
| 2. In the methods and findings in the abstract, it says "Compared to matched controls, before and after adjustment..." but then the presented results - percentages and HR, are they adjusted or unadjusted? It's not clear. | We have now indicated that the HR were adjusted (with the main model selected for the analysis, see below). | P2/L38 |
| 3. The presentation of 6 models is difficult to follow, confusing and redundant. Basically, we only need one ultimate and fully adjusted model as primary analysis, and the rest can go to supplementary information including Figure 2. We want to see the full details of this primary and fully adjusted analysis, not only the main outcomes between those with and without SMI but also the HRs for all those variables adjusted the cox model including all the demorgraphics, case-mix, risk factors and etc. In the way, we may find some clue to explain the findings of this study. | We have now selected a main model and presented the full details of this fully adjusted analysis. We simplified the sensitivity analyses which are now in the supplementary files.  The method section was changed as follows:  “To study the association between each SMI and outcome, the Kaplan–Meier method and the log-rank statistic were used to estimate and compare the cumulative death rates. Hazard ratios (HRs) and 95% confidence intervals (95% CIs) were estimated using Cox survival models with a robust variance estimator to account for clustering within matched pairs. Two models were developed for each outcome. Model 1 included SMI only (no adjustment). Model 2 included SMI with additional covariates of smoking, alcohol, and other substance addiction (yes vs. no), overweight or obesity (yes vs. no), the Charlson comorbidity index (0, 1-2, ≥3), presence of trauma (yes vs. no), surgical intervention (yes vs. no), SAPS II score (modified, without age), organ failures (yes vs. no for each of respiratory, renal, neurologic, cardiovascular, hematologic, metabolic, hepatic), the source of hospital admission (home, transfer from other hospital ward), and time between hospital admission and ICU admission (≤1 vs. > 1 day). The covariates were selected a priori on the basis of clinical relevance or the results of bivariate outcomes analyses (SD>0.2). Interactions with SMI were investigated, but associations were negligible. Several sensitivity analyses were performed: model S1 (model 2 with the 17 Charlson comorbidities instead of the Charlson comorbidity index), model S2 (model 2 with infected organs instead of organ failures), model S3 (model 2 with ICU supportive therapies instead of organ failures), model S4 (model 2 with the nature of isolated pathogens) and model S5 on the whole cohort (without matching process) using the same variables as in model 2 and matching variables to consider residual bias from incomplete matching of controls to the respective SMI group.” | P10/L244 |
| 4. Missing data. What are missing rates for patients' data, variable by variable? How was the missing data issue dealth with in the analyses? The authors didn't mention the missing data at all in the paper, which is inadequate. | A weakness of large hospital databases is the miscoding of diagnoses during hospital stays that can underestimate patients’ comorbidities. Some data is known to be insufficiently coded in medico-administrative databases. Missing data is thus assumed as no presence of disease. However, the coding has been strictly regulated since the DRG system was introduced in France. We have added the following sentence in the limitation section: “Missing data are thus assumed to indicate no disease present. In addition, the key exposure in the present study (*i.e.*, SMI) can be misclassified due to use of ICD-10 codes, which could have affected reported effect estimates. However, the coding has been strictly regulated since the DRG system was introduced in France.”  We propose adding the following limitation in the discussion:  “First, we described only patients who died in hospital, which means that the CFR might be slightly underestimated. However, deaths occurring outside the hospital are extremely rare in France [28]. Our findings at 30 and 90 days were similar to those reported in other studies [69]. In addition, the evolution of the CFR between 30 and 90 days and between 90 and 365 days was similar in the patients with and without SMI, supporting a lack of bias to account for the different extra-hospital mortality.”  28. Armoiry X, Obadia J-F, Pascal L, Polazzi S, Duclos A. Comparison of transcatheter versus surgical aortic valve implantation in high-risk patients: A nationwide study in France. J Thorac Cardiovasc Surg. 2018;156: 1017-1025.e4. doi:10.1016/j.jtcvs.2018.02.092  69. Bauer M et al. Mortality in sepsis and septic shock in Europe, North America and Australia between 2009 and 2019- results from a systematic review and meta-analysis Crit Care. 2020 May 19;24(1):239. | P14/L390  P14/L380 |
| 5. There are miss-matches between case and control, which may help to explain the findings. For table 1 and 2, the comparisons were done using the standardized difference, however it's only one way for comparison. More often we use p-values for comparison. Could authors please use appropirate statistical tests to get p-values for comparison between case and control for the variables? The p-values and SD can be presented side by side. We need to go to the details of the matching proces to see whether it's properly done. | We agree with the Editor and Reviewer 2, we have now added p-values and 95% CI in tables 1 and 2. | Not applicable |
| 6. The authors concluded that "In contrast to the excess mortality from infection observed in patients with SMI, our findings suggested improved outcome from septic shock in the ICU". However, the results are a bit difficult to understand. The interpretation and explanation in the discussion are mainly from some theories and references but not comprehensive and basically not convincing. Maybe worth going back to the study data and investigate the differences in characteristics of the patients with and without SMI and see if it can help the interpretation. | We thank the Reviewer 2 for this major comment. In accordance with this comment and also those of Reviewer 1 and editor, we removed this section of the discussion which was too speculative.  As suggested by the Editor, we added the following explanation: “However, there were some important differences between patients with and without SMI after matching (*e.g.*, fewer malignancies and fewer comorbid conditions), which may have influenced outcomes. Although these differences were adjusted for, it is possible that residual confounding remained. In addition, the social deprivation indicator is based on area-level and may thus also lead to residual confounding.” | P14/L363 |
| 7. 90-day hospital mortality. Does it mean those patients died at hospital by 90 days? What if a patients was discharged before 90-days, I assume there are many, but the subsequently died. Do you have this information and how it's recorded. Basically I'd like to know what is the difference between 90-day hospital mortality and 90-day mortality, and many studies used the latter one. | We have worked on the French National Hospital database, all the patients died in hospital (same stay or rehospitalisation). A unique anonymous identifier enables to link the different inpatient stays of the patients.  In France, the majority of patients die in hospital, reaching about 80% between 60 and 70 years old (the average age of our population). In our study, the findings at 30 and 90 days are similar with those reported in other works. In addition, the evolution of mortality between 30 and 90 days and between 90 and 365 days is similar between the different groups with and without SMI, in favour of the absence of bias caused by a different extra-hospital mortality rate between the groups.  We have added in the method section that “A unique anonymous identifier enables different inpatient stays of individual patients to be linked.”  We have added the following limitation in the discussion:  We propose adding the following limitation in the discussion:  “First, we described only patients who died in hospital, which means that the CFR might be slightly underestimated. However, deaths occurring outside the hospital are extremely rare in France [28]. Our findings at 30 and 90 days were similar to those reported in other studies [69]. In addition, the evolution of the CFR between 30 and 90 days and between 90 and 365 days was similar in the patients with and without SMI, supporting a lack of bias to account for the different extra-hospital mortality.”  28. Armoiry X, Obadia J-F, Pascal L, Polazzi S, Duclos A. Comparison of transcatheter versus surgical aortic valve implantation in high-risk patients: A nationwide study in France. J Thorac Cardiovasc Surg. 2018;156: 1017-1025.e4. doi:10.1016/j.jtcvs.2018.02.092  69. Bauer M et al. Mortality in sepsis and septic shock in Europe, North America and Australia between 2009 and 2019- results from a systematic review and meta-analysis Crit Care. 2020 May 19;24(1):239. | P7/L157  P14/L381 |

**Reviewer 3**

| **Reviewer’s comments** | **Authors’ response and changes made** | **Page/Line** |
| --- | --- | --- |
| 1. This is a review of the manuscript "Severe mental illness is associated with a better prognosis in septic shock: results from 187,587 hospitalizations in France" submitted for publication in PLOS Medicine. This is a very interesting manuscript, which addresses an important question, with potential important therapeutic implications. There is much to like about this manuscript. The results are presented clearly, the methods are sound, the discussion follows well from it, and the manuscript is very well written. Below are my comments and suggestions. | We appreciate the careful reading by Reviewer 3 that gave us the opportunity of improving the quality of the manuscript. | Not applicable |
| 2. As stated by the authors, the reasons for lower septic shock's related mortality in patients with severe mental illnesses (SMI) than in other patients may possibly be linked to their exposures to specific psychotropic medications. I think that the interest in this manuscript would be even more pronounced if secondary exploratory analyses could test the moderating effects of main categories of psychotropic medications in these associations. A complementary approach would be to compare mortality rates between the patients taking psychotropic medications versus not. | We agree with the Reviewer but psychometric medications are not available in our database (PMSI). This issue is highlighted in the limitation section: “There are also limitations associated with the lack of some variables, including specific description of psychotropic medications, body mass index, fitness, blood lactate levels, which could be useful to categorize our patients.“ | P15/L400 |
| 3. Are diagnoses of psychiatric disorders mutually independent or is there a possibility of overlaps? If it is the case, I would present the correlations across disorders. | We thank the reviewer 3 for this important comment. The method section has been completed as follows:  For the purpose of this study, we defined six groups: three groups with SMI, which included patients with a diagnosis of schizophrenia (ICD-10 codes F20*, F22*, or F25*), bipolar disorder (ICD-10 codes F30*, F31*), or major depressive disorder (ICD-10 codes F33*); and three matched groups without SMI (controls). In patients with dual diagnoses, those with codes for schizophrenia and bipolar disorder or major depressive disorder were classified in the schizophrenia group, and those with codes for bipolar disorder and major depressive disorder were classified as bipolar disorder. There was therefore no overlap across the groups.” | P9/L203 |
| 4. I think that the sentence "This hypothesis has been recently reinforced during the COVID-19 pandemic, during which fluoxetine [46] (an antidepressant) and chlorpromazine [47](an antipsychotic) were suggested to have beneficial effects" could be expanded further.  Specifically, a recently published SARS-CoV-2 animal model showed partly independent antiviral and anti-inflammatory effects of fluoxetine (PMID: 36362409), in line with several observational studies (PMID: 36233753; PMID: 35241663; PMID: 36380766). A candidate mechanism, which is shared by several psychotropic medications and is supported by several preclinical (PMID: 34608263) and observational studies (PMID: 36233753; PMID: 35241663; PMID: 35995770; PMID: 34050932), is the Functional Inhibition of Acid Sphingomyelinase (FIASMA). Finally, several RCTs and observational interventional studies have also found evidence of efficacy of fluvoxamine at a daily dose of 200mg or more against COVID-19 among outpatients with COVID-19 (PMID: 33180097; PMID: 34717820; PMID: 35385087) and ICU COVID patients (PMID: 34719789). I think that the inclusion of these data may enrich the discussion. | We thank the Reviewer for this interesting and rich complement that is now added in the discussion as follows:  “Specifically, a SARS-CoV-2 animal model showed independent antiviral and anti-inflammatory effects of fluoxetine [51], in line with several observational studies [52–54]. A candidate mechanism, shared by several psychotropic medications and supported by several preclinical [55] and observational studies [52,53,56,57], is the functional inhibition of acid sphingomyelinase (FIASMA) leading to a regulation of apoptosis, cellular differentiation, proliferation and cell migration. Finally, several RCTs and observational studies have reported evidence of efficacy of fluvoxamine at a daily dose of 200 mg or more against COVID-19 among outpatients with COVID-19 [58–60] and COVID-19 ICU patients [61]. A potential implication of these results is that the frequently observed discontinuation of psychotropic medications on admission to the ICU should be carefully considered given the risks of relapse of the psychiatric disorder as well as the potential benefits of these drugs on mortality in the context of septic shock. Further studies are needed to explore these immune and pharmacological mechanisms.” | P13/L327 |
| 5. Another potential implication of these results is that the frequently observed discontinuation of psychotropic medications in ICUs should be carefully discussed given the risks of relapse of the psychiatric disorder as well as their potential benefits on mortality in the context of septic shock. | Again thank you for this relevant implication that is now added as follows: “A potential implication of these results is that the frequently observed discontinuation of psychotropic medications on admission to the ICU should be carefully considered given the risks of relapse of the psychiatric disorder as well as the potential benefits of these drugs on mortality in the context of septic shock.” | P13/L336 |
