# Supplementary material for: Association of severe mental illness and septic shock case fatality rate in patients admitted to the intensive care unit: A national population-based cohort study
Source: PLoS Med. 2023 Mar 13;20(3):e1004202. doi: 10.1371/journal.pmed.1004202 (PMC10042353; doi:10.1371/journal.pmed.1004202)
Supplement: Attachment — Submitted filename: 2023_02_07_comments on revision 03397R2.docx [file pmed.1004202.s010.docx]

Thank you for sending through the response to reviewer comments and reviewer feedback. I support ‘minor revisions’ but would like to ask the authors to further clarify that their focus is on factors related to the disease process and treatment of SMI. Suggested edits below to clarify this (underlined):

1. The primary objective was to compare the 90-day CFR in septic shock patients with and without SMI admitted to the intensive care unit (ICU), after adjusting for social disadvantage and physical health co-morbidity.”
2. Abstract conclusion: “Our findings suggest that, after adjusting for social disadvantage and physical health co-morbidity, there are improved septic shock outcome in patients with SMI compared to patients without. This finding may be the result of different immunological profiles and exposures to psychotropic medications, which should be further explored.”
3. Limitations: “First, we described only patients who died in hospital, which means that the CFR might be [delete slightly] underestimated. [delete however] Deaths occurring outside the hospital are extremely rare in France but could be differentially experienced by people with SMI [28]. Nonetheless, our findings at 30 and 90 days were similar to those reported in other studies [69]. In addition, the evolution of the CFR between 30 and 90 days and between 90 and 365 days was similar in the patients with and without SMI, supporting a lack of bias to account for the different extra-hospital mortality.”
4. Discussion: “The lower case-fatality rate may have health policy implications on future focus of resource allocation to improve life expectancy in patients with SMI. This finding suggests that the higher mortality rate due to infection/sepsis among patients with SMI reported in previous studies appears to be due to the increased risk of infection/sepsis among patients with SMI and potentially poorer access to timely and adequate care, but not due to greater case fatality once they have [delete developed] been hospitalised for septic shock.”

In addition, please can the authors clarify how many people die in the first 48 hours of ICU admission and add this as a potential limitation due to the risk of misclassification (which could be differential with respect to SMI status).
