# Supplementary material for: Association of severe mental illness and septic shock case fatality rate in patients admitted to the intensive care unit: A national population-based cohort study
Source: PLoS Med. 2023 Mar 13;20(3):e1004202. doi: 10.1371/journal.pmed.1004202 (PMC10042353; doi:10.1371/journal.pmed.1004202)
Supplement: Attachment — Submitted filename: ResponseLetter PLOSMED 14022023.docx [file pmed.1004202.s011.docx]

**Editor**

| **Editor’s comments** | **Authors’ response and changes made** | **Page/Line** |
| --- | --- | --- |
| GENERAL  Thank you for your detailed and considerate responses to previous editor and reviewer comments, which we appreciate and accept. Please see below for further minor revisions which we require that you address in full. | Dear Editor,  We thank you for your comments and your questions. Please find our response below. | Not applicable |
| AUTHOR SUMMARY  Thank you for including an author summary which reads very nicely – please see reviewer comments below for suggested revisions. | We thank you for your time and attention, and we are grateful that you give us the opportunity to correct and improve our manuscript. | Not applicable |
| FIGURES  To make your figures more accessible to those with colour blindness, please consider avoiding the use of red (or green). | We thank you for this suggestion, the colours have now been changed. | Not applicable |
| SOCIAL MEDIA  To help us extend the reach of your research, please provide any Twitter handle(s) that would be appropriate to tag, including your own, your coauthors’, your institution, funder, or lab. Please detail any handles you wish to be included when we tweet this paper, in the manuscript submission form when you re-submit the manuscript. | The Twitter handles that should be tagged are the following: @MarcLeone8 @GuillaumeFond @LakbarInes @univamu @aphm_actu | Not applicable |
| Thank you for sending through the response to reviewer comments and reviewer feedback.  I support ‘minor revisions’ but would like to ask the authors to further clarify that their focus is on factors related to the disease process and treatment of SMI. Suggested edits below to clarify this (underlined):  The primary objective was to compare the 90-day CFR in septic shock patients with and without SMI admitted to the intensive care unit (ICU), after adjusting for social disadvantage and physical health co-morbidity.” | We have added the sentence as wisely suggested. We are hopeful that the meaning is now clear. | Page 3, line 45 |
| Abstract conclusion: “Our findings suggest that, after adjusting for social disadvantage and physical health co-morbidity, there are improved septic shock outcome in patients with SMI compared to patients without. This finding may be the result of different immunological profiles and exposures to psychotropic medications, which should be further explored.” | We thank you for this suggestion. The modification has been done accordingly | Page 4, line 73 |
| Limitations: “First, we described only patients who died in hospital, which means that the CFR might be [delete slightly] underestimated. [delete however] Deaths occurring outside the hospital are extremely rare in France but could be differentially experienced by people with SMI [28]. Nonetheless, our findings at 30 and 90 days were similar to those reported in other studies [69]. In addition, the evolution of the CFR between 30 and 90 days and between 90 and 365 days was similar in the patients with and without SMI, supporting a lack of bias to account for the different extra-hospital mortality.” | Thank you for your suggested amendments. The modifications have been made accordingly. | Page 13, line 349 |
| Discussion: “The lower case-fatality rate may have health policy implications on future focus of resource allocation to improve life expectancy in patients with SMI. This finding suggests that the higher mortality rate due to infection/sepsis among patients with SMI reported in previous studies appears to be due to the increased risk of infection/sepsis among patients with SMI and potentially poorer access to timely and adequate care, but not due to greater case fatality once they have [delete developed] been hospitalised for septic shock.” | The modification has been done accordingly. | Page 13, line 336 |
| In addition, please can the authors clarify how many people die in the first 48 hours of ICU admission and add this as a potential limitation due to the risk of misclassification (which could be differential with respect to SMI status). | We thank the editor for this comment. As explained in the method section, we limited inclusion to patients with an ICU length of stay of at least 48 h, unless the patient died within 48 h, in order to avoid over-estimating diagnoses of septic shock. So patients who died were included in the first 48 hours of ICU admission and there is no risk of misclassification.  As asked, please find the data on deaths in the first 48 hours of ICU admission: a total of 9,352 died in the first 48 hours of ICU admission: 8,922/176,271 for patients without SMI (5.06%), patients with schizophrenia 139/3,812 (3.65%), patients with bipolar disorder 92/2,258 (4.07%) and patients with major depressive disorder 199/5,246 (3.79%). | Not applicable |

**Reviewer 1**

| **Reviewer’s comments** | **Authors’ response and changes made** | **Page** |
| --- | --- | --- |
| Reviewer #1: The authors have addressed systematically the vast majority of the comments, with extensive revision of the manuscript. Few issues remain. | We appreciate the careful reading of Reviewer 1 that gave us the opportunity to improve the quality of the manuscript. We hope that these improvements will bring this manuscript to the level of your expectations. | Not applicable |
| 1. The clinical/research relevance of reporting a study on a patient group with lower (rather than higher) case fatality among septic patients was addressed only in a narrow fashion (e.g., need to exercise care when considering discontinuation of psychotropics on ICU admission).  Specifically, a key long-term goal of identifying patient groups with higher case fatality in sepsis than that in the general population (as commonly reported) is (beyond providing clinicians data for prognostication and other bedside decision-making) to identify the mechanisms underlying the outcome differences and, critically, modifiable mechanisms that can serve as targets for interventional approaches geared to reduce the outcome disparity of the affected group in reference to the general population.  On the other hand, the present study demonstrates an unexpectedly lower case fatality in a patient group (severe mental disorders) that would be expected to have higher case fatality, where the studied group is, critically, well-documented to have immune dysfunction across multiple domains at baseline. Thus, a key implication of this study is that future work to characterize potential differences in response to infection among patients with and without severe mental disorders across key domains of the immune system may identify potential targets for therapeutic interventions to reduce short-term mortality in the general population. Adding these implications to the Discussion can provide readers with broader context for the study's findings. | We thank you for this relevant comment. The following sentence has now been added to the discussion section: “The long-term goal of identifying patient groups with higher case fatality in sepsis than that in the general population is to identify the mechanisms underlying the outcome differences and, critically, modifiable mechanisms that can serve as targets for interventional approaches geared to reduce the outcome disparity of the affected group in reference to the general population.” | Page 12 line 327 |
| 2. The authors have added, as recommended, to the study limitations the potential of miscoding of mental disorders and lack of data on processes of care across examined groups in their cohort. Addressing some key implications of these limitations can add further context to the study findings.  Specifically, misclassification of mental disorders would be expected to blur the differences between groups and thus diminish outcome differences between septic shock patients with and without mental disorders; this would suggest that the study's findings may represent possible underestimation of the magnitude of the better outcomes observed among patients with mental disorders.  Similarly, as related to unknown and potentially different processes of care between septic shock patients with and without severe mental disorders, patients with mental disorders are well-documented to receive poorer quality of healthcare, and stigma, stereotyping, and negative attitudes towards these patients  by clinicians appear prevalent. Such care differences would be not be expected, however, to result in better outcomes of septic patients with mental disorders; indeed, as noted for potential miscoding of mental disorders, such potential differences in care processes would suggest that the study's findings may represent possible underestimation of the magnitude of the better outcomes observed among patients with mental disorders. | The following sentence has been added to the discussion section as wisely suggested: “Misclassification of mental disorders would be expected to blur the differences between groups and thus diminish outcome differences between septic shock patients with and without SMI. This would suggest that the study's findings may represent possible underestimation of the magnitude of the better outcomes observed among patients with SMI".  We thank reviewer 1 for bringing this to our attention and correcting a potentially significant omission. The following sentence has been added to the discussion section: “Patients with SMI are well-documented to receive poorer quality of healthcare, in addition to stigma, stereotyping, and negative attitudes towards these patients by clinicians. Such care differences would be not be expected, however, to result in better outcomes of septic patients with SMI. Such potential differences in care processes would suggest that the study's findings may represent possible underestimation of the magnitude of the better outcomes observed among patients with SMI.” | Page 14 line 371  Page 14 line 391 |
| 3. In the Abstract, under Background, the revised last part of the first sentence is confusing (repeated use of "associated"). Revising that part to read "but whether SMI is associated with higher or lower case fatality rates (CFRs) among infected patients remains unclear" would improve clarity and focus. | Thank you noticing this and amending an important and potentially misleading oversight. The modification has been done accordingly | Page 3 line 45 |
| 4. In the Author Summary, under the section "What do these findings mean?" I would suggest including a brief notation that the findings also indicate that the excess mortality from sepsis (as noted under "Why was this study done?") is due to an increased risk of sepsis/infection among patients with severe mental disorders, but not due to increase case fatality among septic patients. | “Our findings also suggest that the excess mortality from sepsis is due to an increased risk of sepsis/infection among patients with SMI, but not due to increase case fatality among septic patients” | Page 5 line 104 |

**Reviewer 2**

| **Reviewer’s comments** | **Authors’ response and changes made** | **Page/Line** |
| --- | --- | --- |
| Reviewer #2: Thanks authors for their great effort to improve the manuscript. All my comments were well addressed in a professional way. I am satisfied with the response and revision. No further issues needing attention. | We appreciate the careful reading of Reviewer 2 that gave us the opportunity to improve the quality of the manuscript. | Not applicable |

**Reviewer 3**

| **Reviewer’s comments** | **Authors’ response and changes made** | **Page/Line** |
| --- | --- | --- |
| The authors did a great job in responding to my comments.  I thank them and congratulate them for this well-designed and important study. | We appreciate the careful reading of Reviewer 3 that gave us the opportunity to improve the quality of the manuscript. | Not applicable |
